# Nanomedicine-enabled disruption of glucose metabolism and synergistic antitumor therapy

**DOI:** 10.1186/s12951-025-03984-w

**Published:** 2026-03-07

**Authors:** Jiang Ni, Ang Ma, Qiufang Gao, Chenxu Li, Dan Li, Rong Wang, Yang Ding, Hong Cao

**Affiliations:** 1 Department of Pharmacy & Department of Nutrition, Affiliated Hospital of Jiangnan University, Wuxi, China; 2 Wuxi School of Medicine, Jiangnan University Medical Center, Jiangnan University, Wuxi, China; 3 Department of Pharmaceutics, China State Key Laboratory of Natural Medicines, China Pharmaceutical University, Nanjing, China; 4 State Key Laboratory for Quality Ensurance and Sustainable Use of Dao-di Herbs, Artemisinin Research Center, Institute of Chinese Materia Medica, China Academy of Chinese Medical Sciences, Beijing, 100700 China

**Keywords:** Nanomedicine, Drug delivery, Glucose metabolism reprogramming, Combination therapy, Antitumor therapy

## Abstract

Tumor cells exploit glucose metabolic reprogramming, most notably the Warburg effect of aerobic glycolysis to fuel rapid proliferation, biosynthetic precursor production, and immunosuppressive microenvironment formation. While this creates therapeutic vulnerabilities, conventional metabolic inhibitors face critical limitations in systemic toxicity, metabolic heterogeneity, and inadequate tumor accumulation. Hence, this review details how nanomedicines overcome these limitations through active targeting, enhanced tumor accumulation, stimuli-responsive drug release, and co-delivering multiple drugs for combination therapy. Guided by the glucose metabolic cascade, we comprehensively discuss nano-enabled strategies to achieve antitumor efficacy through inhibiting glucose transportation, blocking glucose supply, accelerating glucose depletion, suppressing glycolytic enzymes, and concurrently inhibiting oxidative phosphorylation. Furthermore, this review summarizes strategies leveraging nanomedicines to integrate glucose metabolism modulation with complementary therapies, such as immunotherapy, radiochemotherapy, cuproptosis induction, and photothermal therapy, for synergistically eradicating tumors. Recognizing glucose metabolism reprogramming as a pivotal therapeutic target in oncology, we emphasize the indispensable role of nanomedicine in overcoming persistent barriers to advance tumor-selective metabolic intervention, thereby elevating treatment efficacy and accelerating the clinical translation of precision nanotherapeutics.

**Graphical abstract:**

**Figure:**
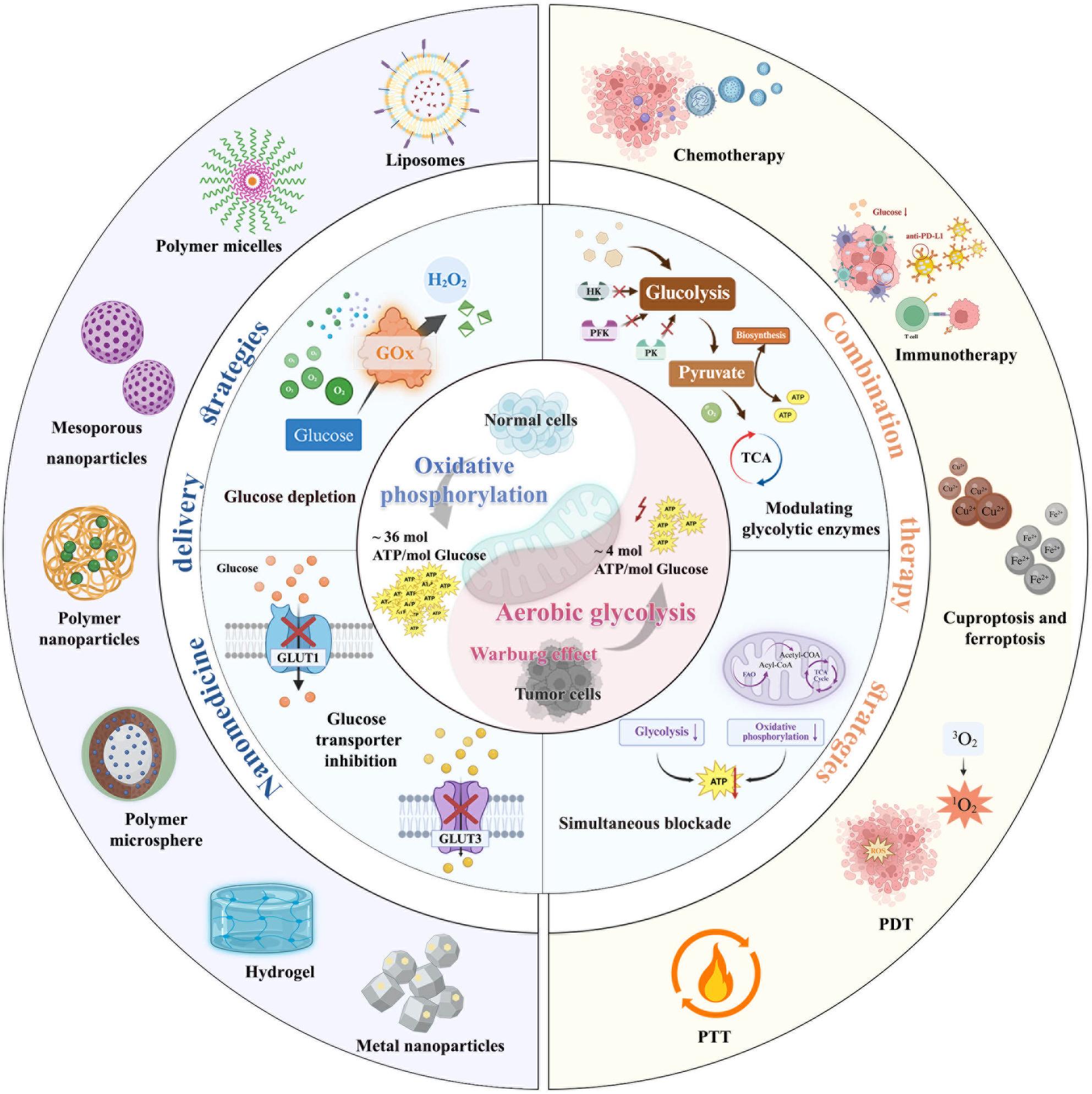
Graphical Abstract

## Background

 In contrast to normal cells, tumor cells exhibit profound dysregulation of glucose metabolism to meet the bioenergetic and biosynthetic demands of rapid proliferation [1, 2]. Even under aerobic conditions, tumor cells mainly metabolize glucose through glycolysis, generating lactic acid and releasing energy, which is known as Warburg Effect [3, 4]. When compared with oxidative phosphorylation, this glucose metabolic reprogramming favors inefficient but rapid adenosine triphosphate (ATP) generation through glycolysis, which not only correlates with tumor aggressiveness and poor prognosis but also represents a critical driver of malignant progression [5, 6]. Beyond fueling rapid proliferation through accelerated ATP production, tumor cells exploit glucose metabolic reprogramming to divert glycolytic intermediates, including 3-phosphoglycerate and pentose phosphate pathway products into biosynthesis of nucleotides, lipids, and amino acids essential for rapid proliferation [7–9]. Moreover, this metabolic plasticity extends to mitochondrial adaptation, where certain aggressive subpopulations (e.g., metastatic cells) maintain flexible oxidative capacity to survive microenvironmental stresses, demonstrating the multifaceted role of metabolic rewiring in tumor progression [10]. The resulting dependence on glycolytic flux renders cancer cells particularly vulnerable to glucose deprivation, positioning glucose metabolic intervention as an emerging therapeutic strategy in oncology.

Beyond fueling tumor cell rapid proliferation, glucose metabolic reprogramming dynamically interfaces with diverse antitumor modalities to dictate treatment responsiveness [11, 12]. For example, glycolysis-derived ATP fuels deoxyribonucleic acid (DNA) damage repair, leading to chemotherapy-resistance and radioresistance [13, 14]. Concurrently, glycolysis generates bioactive intermediates that activate tumor anti-apoptotic pathways and exacerbate hypoxia, collectively diminishing the efficacy of radio- and chemotherapy [15]. And, unlike conventional radio-chemotherapy, tumors exhibit more complex crosstalk with emerging immunotherapies [16, 17]. Specifically, aberrant glucose metabolism in cancer cells not only establishes an immunosuppressive microenvironment but also instigates glucose competition with immune cells [18]. More specifically, hyperactive glucose uptake by tumor cells depletes extracellular glucose in the tumor microenvironment, inducing insufficient bioenergy in T cells and impairing immune activation. Moreover, lactate derived from glycolysis serves as a key immunosuppressive effector, dysregulating functions of T cells, macrophages, and other immune cells [19, 20]. The high glycolytic flux in aggressive tumors also heightens susceptibility to copper ionophores by disrupting mitochondrial metabolism, while glucose deprivation synergizes with ferroptosis/pyroptosis inducers by depleting glutathione and ATP [21, 22]. A metabolic-therapeutic paradox exists between glucose metabolism and photodynamic therapy: tumor glycolysis fuels reactive oxygen species (ROS)-scavenging antioxidants while simultaneously depleting oxygen essential for photodynamic therapy (PDT) efficacy [23]. In addition, tumor metabolic heterogeneity poses dual challenges where oxidative phosphorylation-dependent subpopulations evade glycolysis inhibitors, necessitating combined pathway blockade [24, 25]. Meanwhile, spatiotemporal mismatches between metabolic modulators and secondary therapies remain a critical barrier for nanotherapeutics. Considering the complex crosstalk between glucose metabolism regulation and other antitumor strategies, in-depth clarification of their crosstalk mechanisms and proposal of appropriate combination strategies are expected to improve antitumor efficacy.

However, conventional approaches to disrupt tumor glucose metabolism, including small-molecule inhibitors of glycolysis, glucose transporters, or mitochondrial pathways, face significant challenges that limit their clinical efficacy [26, 27]. These limitations primarily stem from systemic toxicity, poor pharmacokinetics, and inadequate tumor penetration, compounded by the metabolic plasticity of cancer cells [28, 29]. Since members of the glucose transporter (GLUT) family, such as GLUT1 and GLUT3, are widely expressed in normal tissues, including the brain and red blood cells, non-selective inhibition of their activities can lead to systemic hypoglycemia and tissue energy depletion. In addition, glycolysis inhibitors (e.g., 2-deoxyglucose, 2-DG) disrupt glucose metabolism in highly glycolytic normal tissues, such as the brain (neurotoxicity) and heart (cardiotoxicity) [30]. Worse still, most drugs not only suffer from issues such as low solubility and rapid in vivo clearance but also struggle to penetrate into tumor due to multiple physiological barriers within the tumor microenvironment [31].

Nanomaterials have emerged as transformative platforms for cancer treatment by overcoming critical limitations of conventional therapies [32]. For disrupting tumor glucose metabolism, nanomaterials share merits in active targeting via surface modifications, improving drug accumulation within the tumor, on-demand drug release through stimuli-responsive designs according to endogenous stimulating signals, such as pH, ROS, and enzymes, and multifunctionality integrating diagnostics with combination therapies [33–35]. Moreover, through well-designed, nanomaterials can remodel tumor microenvironment to enhance anti-tumor efficacy [36, 37]. These unique properties of nanomaterials make them ideally suited for glucose metabolism intervention therapies, effectively addressing the limitations of conventional approaches such as poor targeting specificity, off-target toxicity, and uncontrolled drug release [38, 39].

**Fig. 1:**
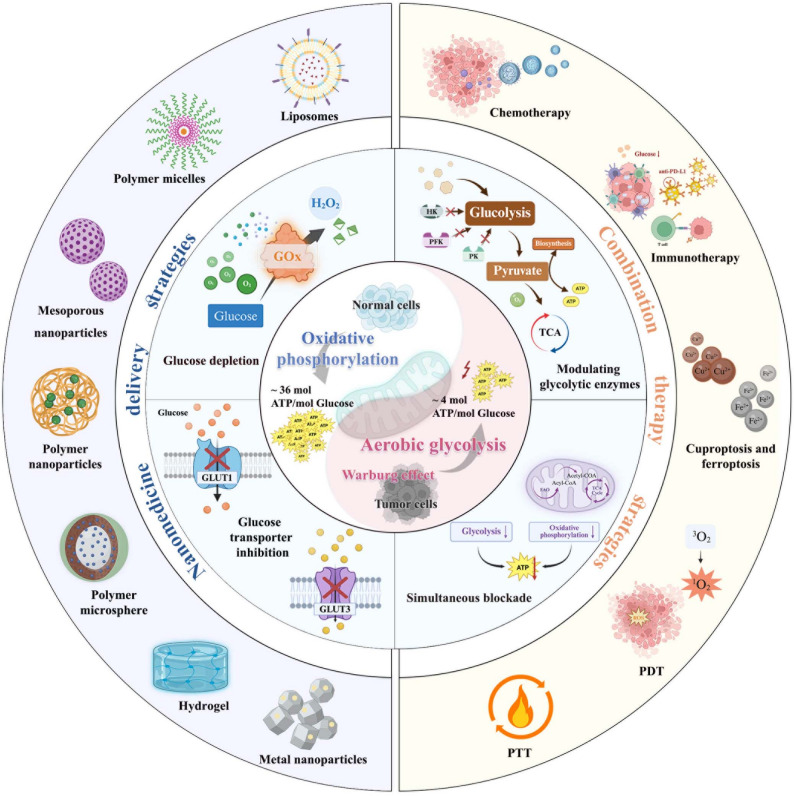
Schematic illustration of nanomedicines interfering with glucose metabolism for tumor therapy. An overview of glucose metabolism dysfunction in tumors, “glucose metabolic cascade” inspired modulating strategies in anti-tumor therapy, representative nanomedicines, and combination treatment strategies

For nanomaterials-enhanced glucose metabolism intervention, nanotechnology provides innovative solutions to modulate tumor glucose metabolism by blocking glucose uptake, inhibiting glucose transporter [40, 41], glucose depletion [42–44], modulating glycolytic enzymes [45, 46], reprogramming metabolic pathways [47], and combining glucose metabolic regulation with other therapies [48, 49]. By overcoming metabolic plasticity through dual-pathway inhibition, nanoplatforms represent a paradigm shift in precision cancer metabolism therapy [50]. Hence, this review highlights how glucose metabolic reprogramming drives tumor progression by fueling proliferation, immune evasion, and therapy resistance while emphasizing nanomaterial-based strategies to precisely target these pathways (Fig. 1). By overcoming limitations of conventional therapies, nanomedicines enable spatiotemporally controlled intervention through glycolysis inhibition, combinatorial metabolic-immune therapies, and the combination therapy of glucose metabolic with other treatment strategies, offering transformative potential for cancer treatment.

## Strategies of nanomedicine-mediated glucose metabolism modulation

**Fig. 2:**
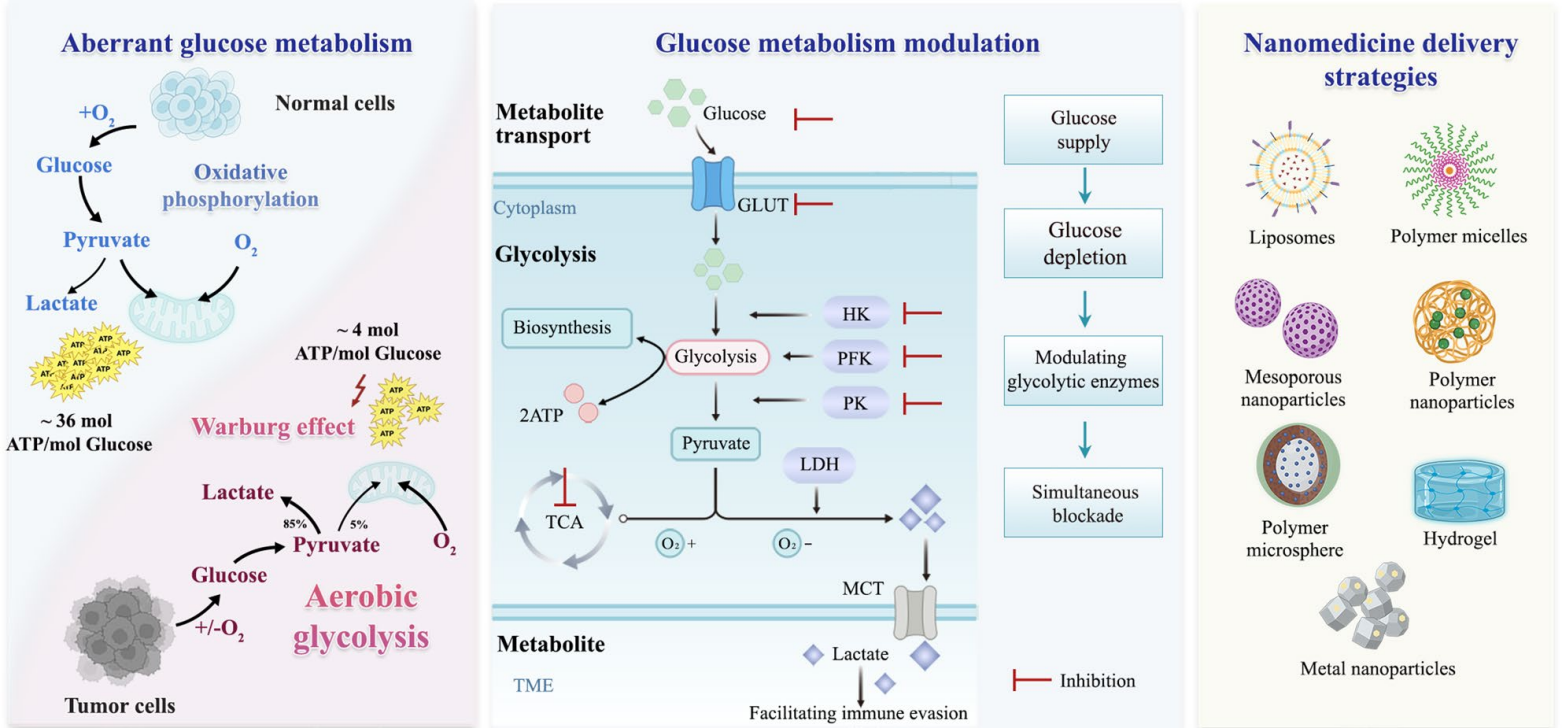
Schematic illustration of aberrant glucose metabolism of tumor cells, the glucose metabolic circuitry that encompasses sequential processes of glucose uptake, intracellular transport, depletion, and utilization, which include regulatory strategies for glycolytic enzymes to optimize glucose utilization. And, the representative nanomedicine that could modulate glucose metabolism for tumor therapy

Aberrant glucose metabolism drives tumor cells toward aerobic glycolysis, enhancing glucose uptake and lactate production. This metabolic reprogramming provides both energy and biosynthetic precursors to fuel tumor cell growth, proliferation, invasion, and metastasis, thereby accelerating oncogenesis [51, 52]. Furthermore, dysregulated glucose metabolism modulates tumor biology through multifaceted mechanisms, including remodeling the tumor microenvironment, deregulating signaling pathways, and facilitating immune evasion [53]. It critically promotes tumor progression by creating a permissive niche for malignant cell survival and expansion [54]. Consequently, as shown in Fig. 2, rational engineering of nanomedicines that precisely target glucose metabolism, which includes glucose supply, glucose depletion, modulation of glycolytic enzymes, and simultaneously blocking oxidative phosphorylation and glycolysis, represents a promising therapeutic paradigm to disrupt tumor bioenergetics and halt malignant progression [55, 56].

### Nanomedicine designed for blocking the supply of glucose

The bioenergetic paradox arising from inefficient ATP generation via aerobic glycolysis and the proliferative urgency of tumor cells compels malignant cells to escalate glucose dependency. The reprogramming of glucose metabolism, a hallmark of cancer, positions glucose uptake inhibition as a selective therapeutic strategy to exploit the metabolic addiction of tumors [57]. Central to this process are glucose transporters (e.g., GLUT1, GLUT3), which are frequently overexpressed in malignant cells to sustain their hyper-glycolytic phenotype and meet the elevated bioenergetic demands of proliferation and survival [58, 59]. Current mainstream strategies to reduce tumor glucose uptake primarily involve inhibition of glucose transporters, which can be employed as monotherapy or in combination. However, these approaches face critical delivery challenges, including poor tumor penetration, rapid systemic clearance, and off-target toxicity, necessitating nanomaterials-mediated targeted delivery systems to achieve tumor-specific accumulation and enhanced therapeutic precision.

#### Nanomedicine targeting GLUT1 to block glucose supply

As a key transporter responsible for transmembrane glucose flux in tumor cells, GLUT1 is synthesized in the rough endoplasmic reticulum [60]. Following post-translational modifications (e.g., N-glycosylation) in the Golgi apparatus, it is trafficked via vesicular transport to the plasma membrane [61]. There, it facilitates glucose uptake through energy-independent facilitated diffusion, fueling the bioenergetic demands of rapid tumor proliferation [62]. Consequently, targeting GLUT1 biogenesis, maturation, or functional expression represents a promising therapeutic strategy to disrupt glucose supply in malignancies.

Intriguingly, capitalizing on the finite membrane distribution of GLUT1, the researchers engineered glucose-decorated nanoparticles that competitively bind GLUT1 to starve tumor cells by impeding glucose uptake [63]. These nanoparticles were fabricated from chitosan and poly(lactic-co-glycolic acid), with surface-conjugated glucose enabling dual functionality of GLUT1-mediated tumor targeting and competitive inhibition of glucose import [64]. In HT-29 cells treated with glucose-decorated nanoparticles, cell viability plummeted to 47%, demonstrating potent metabolic disruption. However, this study was only conducted in vitro cell experiments to evaluate the therapeutic effect and did not take into account the complexity of the in vivo environment. In particular, glucose-modified nanoparticles may also block the glucose uptake of other normal cells, especially highly energy-consuming cells such as brain cells, potentially causing adverse side effects.

Considering the crucial roles of Golgi apparatus in GLUT1 generation, Li et al. engineered a Golgi apparatus-targeted nanodelivery system that disrupts GLUT1 trafficking and glycolytic flux, thereby inducing tumor-specific metabolic starvation [65]. Tellurium-doped nanodiamonds encapsulated with human serum albumin were engineered as a photothermally activatable nanoplatform to enable spatiotemporally controlled metabolic intervention [66]. Upon near-infrared irradiation (NIR), the system achieved on-demand apigenin release, while tellurate ions simultaneously disrupted Golgi-mediated GLUT1 trafficking, leading to suppressed glucose uptake. This dual interference was further reinforced by apigenin-mediated pyruvate kinase M2 (PKM2), resulting in coordinated blockade of glycolytic flux at both transporter and enzymatic levels. In vivo, this cascade-regulated metabolic inhibition produced potent tumor suppression with minimal systemic toxicity, underscoring the advantage of integrating photothermal control with multi-level metabolic targeting.

Through techniques such as molecular docking, researchers screened out BAY-876 as a highly selective GLUT1 inhibitor. BAY-876 can specifically bind to GLUT1, inhibit its transport function, reduce the glucose uptake of tumor cells, thereby interfering with the energy metabolism and biosynthesis processes of tumor cells. Additionally, BAY-876 also has the potential to induce oxidative stress to kill tumor cells and regulate the immunosuppressive tumor microenvironment. However, its application faces issues such as poor tumor site selectivity and insufficient action specificity. To overcome these limitations, Ma et al. developed an albumin-based nanoplatform to simultaneously disrupt glucose and glutamine metabolism, addressing metabolic compensation associated with single-pathway inhibition [67].

**Fig. 3:**
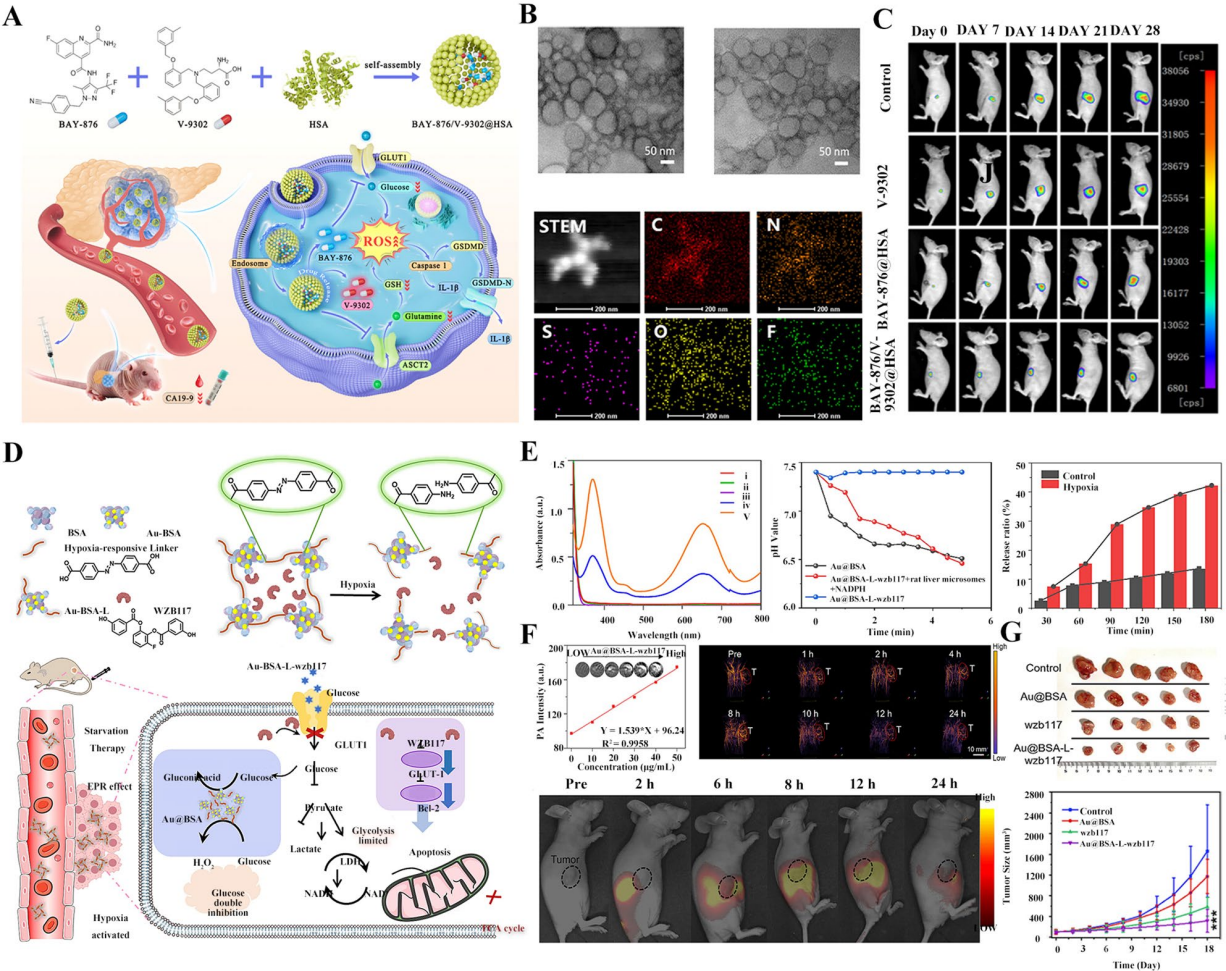
Nanomedicine targeting GLUT1 to block glucose supply. (**A**) Schematic diagram of BAY-876/V-9302@HSA design, preparation, in vivo transport, and action mechanism. (**B**) BAY-876/V-9302@HSA exhibited a spherical-like morphology, and the distribution diagram of various elements on the nanomedicine was as presented. (**C**) The anti-tumor efficacy of BAY-876/V-9302@HSA on tumor-bearing nude mice [67]. Copyright © 2024 American Chemical Society. (**D**) Schematic diagram of Au-BSA-L-WZB117 design, preparation, in vivo transport, and action mechanism. (**E**) TMB assay, changes of pH value, and drug release profiles of Au-BSA-L-WZB117. (**F**) The tumor targeting capability of Au-BSA-L-WZB117 on tumor-bearing nude mice. (**G**) The anti-tumor efficacy of Au-BSA-L-WZB117 on tumor-bearing nude mice. ***p < 0.001 [67]. Copyright © 2024 American Chemical Society

By exploiting hydrophobic domain-drug interactions, the GLUT1 inhibitor BAY-876 and the glutamine uptake inhibitor V-9302 were co-assembled into human serum albumin (HSA) nanoparticles (Fig. 3A), enabling pH-responsive lysosomal release of BAY-876 for effective glucose transport blockade. The BAY-876/V-9302@HSA nanoparticles were spherical in shape (Fig. 3B). This dual-substrate deprivation strategy induced pronounced energy collapse in tumor cells while benefiting from the favorable tumor accumulation and biocompatibility of albumin carriers. In an orthotopic pancreatic cancer model, this metabolic co-inhibition approach resulted in robust tumor suppression with minimal systemic toxicity (Fig. 3C), highlighting the advantage of co-targeting parallel nutrient pathways to overcome metabolic plasticity.

Moreover, Liu et al. synthesized a degradable sonodynamic pseudo-conjugated polymer (SPCP) and a cystine-containing polymer (CCP), and co-assembled them with the BAY-876 to obtain the SPCP/CCP@Bay nanomedicine [68]. Upon ultrasonic activation, the nanocarrier released both BAY-876 and cystine, achieving on-demand metabolic disruption combined with redox modulation. This ultrasound-triggered metabolic intervention not only suppressed primary tumor growth but also enhanced systemic antitumor immunity when combined with α-PD-1 therapy, leading to pronounced inhibition of both local and distant tumors. This work highlights the potential of externally activatable nanomedicines to synchronize metabolic blockade with immunotherapy for improved control of metastasis and recurrence.

As an irreversible inhibitor of GLUT1, WZB117 plays a critical role in tumor starvation therapy. Leveraging this property, Mou et al. adsorbed WZB117 onto hypoxia-responsive nanoparticles (Au@BSA-L) to form the hypoxia-responsive nanomedicine Au@BSA-L-WZB117 [67]. As shown in Fig. 3D, using bull serum albumin (BSA)-stabilized gold nanozyme (Au-BSA) as the core, a hypoxia-responsive linker was crosslinked to the amino groups on the BSA surface, followed by encapsulation of WZB117 to construct the nanomedicine Au-BSA-L-WZB117. Upon reaching the hypoxic tumor region, the azo bond cleavage triggered Au-BSA-L-WZB117 disassembly, leading to burst release of WZB117 to inhibit GLUT1 transport activity. In vitro 3,3’,5,5’-Tetramethylbenzidine (TMB) colorimetric assay for H₂O₂ generation and pH change detection showed that the Au-BSA-L-WZB117 enabled hypoxia-responsive drug release, promoted H₂O₂ production, and induced local acidification, which could be further utilized for responsive drug release (Fig. 3E). Verified by both hotoacoustic imaging and living imaging in Fig. 3F, targeted delivery of Au-BSA-L-WZB117 was achieved by taking advantage of the properties of albumin nanoparticles, which could easily penetrate the vascular endothelium and accumulate at the tumor site via the EPR effect. In a nude mouse orthotopic pancreatic cancer xenograft model, Au-BSA-L-WZB117 significantly inhibited tumor growth and prolonged survival time (Fig. 3G).

Gene silencing technologies, particularly RNA interference (RNAi), enable the specific and efficient downregulation of target protein and receptor expression, holding promise for suppressing GLUT1 expression. However, their clinical translation is limited by challenges in intracellular delivery to target cells and the potential for off-target gene silencing effects in non-target tissues. By leveraging the drug delivery advantages of gold nanoparticles (AuNPs), Li et al. developed a nucleic acid-modified nanomedicine (AuNPs@anti-miR-21/siGlut1), loaded with microRNA-21 (miR-21) inhibitor and GLUT1-targeting siRNA (siGlut1). This nanomedicine utilized miR-21-triggered toehold-mediated strand displacement to achieve efficient delivery of siGlut1. In an A549 tumor-bearing mouse model, the treatment group exhibited an approximately 50% reduction in tumor volume compared to the control group, with no significant systemic toxicity (e.g., body weight changes or organ damage) [69].

While GLUT1 inhibition holds promise for targeting tumor metabolism, it is important to address several clinical concerns related to its therapeutic application. As GLUT1 is highly expressed in erythrocytes, its non-selective inhibition would impair glucose uptake in red blood cells, disrupting their energy metabolism and leading to reduced oxygen transport capacity. This may result in anemia, which could pose a substantial risk to patients undergoing GLUT1-targeted therapies. Moreover, the inhibition of GLUT1 expression in the BBB could compromise the glucose supply to the brain, potentially leading to neurotoxic effects. This is particularly relevant for brain tumors or therapies involving systemic drug delivery to brain tissue, as the disruption of glucose homeostasis in the brain could lead to cognitive deficits or other neurological side effects.

#### Nanomedicine targeting GLUT3 to block glucose supply

Functioning as another glucose transporter, GLUT3 exhibits a distinct expression profile from the more ubiquitously distributed GLUT1. GLUT3 is predominantly localized on neuronal and cardiomyocyte membranes, where it facilitates energy metabolism in these cells. Notably, in the context of anti-tumor therapy, GLUT3 is highly expressed in highly aggressive tumors, particularly brain tumors, making it a promising therapeutic target for glioblastoma and brain metastases. For targeting GLUT3, GLUT3 siRNA delivery was achieved using cationic lipid-assisted PEG-PLA polymeric nanoparticles, targeting tumor cells and glioma stem cells overexpressing GLUT3 [56]. Treatment with this nanomedicine (NP_siRNA_) reduced glucose uptake in tumor cells by approximately 50% and significantly increased ROS levels. In the U87MG tumor-bearing mouse model, intravenous administration of the NP_siRNA_ significantly inhibited tumor growth. It concurrently downregulated GLUT3 expression and reduced levels of stemness-associated genes within tumor tissues, accompanied by a decreased proportion of CD133 cells. Notably, no significant change in mouse body weight was observed, demonstrating low systemic toxicity.

Moreover, Pan et al. developed a dual-targeting liposomal nanoplatform co-loading paclitaxel and glycosylated ginsenoside Rg3 to simultaneously modulate tumor glucose metabolism and the immunosuppressive microenvironment [70]. Through glucose-mimicking surface glycosylation, the nanocarrier achieved selective uptake by tumor cells via GLUT1 and bone marrow-derived suppressor cells (MDSCs) via GLUT3, respectively, enabling concurrent chemotherapy and metabolic suppression of immunosuppressive myeloid cells. This coordinated targeting strategy significantly amplified antitumor efficacy by reshaping glucose-dependent immune suppression, while maintaining favorable systemic safety, as validated in both tumor-bearing mice and non-human primates.

Although there are currently limited studies on GLUT3-targeted anti-tumor therapy, its higher affinity for glucose, relatively specific distribution in tumor cells, and greater sensitivity to tumors in a low-glucose microenvironment (such as the necrotic area) endow GLUT3-targeted anti-tumor therapy with a more promising therapeutic prospect, especially for glioblastoma, brain metastases, and chemotherapy-resistant tumors. However, GLUT3-targeted therapy has limitations in application, including potential neurotoxicity and restrictions imposed by the blood-brain barrier in most treatment scenarios. In the future, the blood-brain barrier (BBB) penetration issue of GLUT3 inhibitors can be overcome by developing BBB-permeable nanomedicine, and non-specific neurotoxicity can be reduced by designing tumor microenvironment-responsive (such as pH/ROS-responsive) intelligent nanomedicines.

**Table 1:** Summary of nanomedicine strategies targeting glucose metabolism

**Therapeutic target**	**Nanoparticles**	**Animal model**	**Administration route**	**Treatment duration**	**Dose (mg/kg)**	**Quantitative outcome (with p-value)**	**Ref**
GLUT1	Tellurium-doped nanodiamonds	4T1 tumor-bearing BALB/c mice	Intravenous injection	15 Days (Day 0, 3, 6, 9, 12 TAH + NIR)	10.0 mg/kg (TAH)	77.8% Tumor growth inhibition rate (p<0.001 vs control)	[65]
	Human bovine serum albumin nanoparticles	Orthotopic pancreatic xenograft tumor-bearing nude BALB/c mice	Intravenous injection	28 Days (Day 0, 2, 4)	7.0 mg/kg (BAY-876) and 2.3 mg/kg (V9302)	N/A Reduced tumor volume rate (p<0.001 vs control)	[119]
	Degradable sonodynamic pseudo-conjugated polymer nanoparticles	MB49 bladder tumor-bearing C57BL/6 mice	Intravenous injection	12 Days (Day 0, 2, 5, 8 (SPCP/CCP@Bay); Day 1, 3, 6, 9 (Ultrasound radiation))	2.5 mg/kg (BAY)	89.1% Tumor inhibition rate (p<0.001 vs control)	[68]
	Hypoxia-responsive nanoparticles	MCF-7 tumor-bearing mice	Intravenous injection	14 Days (Day 0, 2, 4, 6, 8,10)	2 mg/kg (Au@BSA) and 1 mg/kg (wzb117)	N/A Inhibited tumor growth (p<0.001 vs control)	[67]
	Nucleic acid-modified gold nanoparticles	A549 tumor-bearing nude BALB/c mice	Orthotopic injection	/	/	N/A Reduced tumor volume (p<0.01 vs control)	[69]
GLUT3	Lipid-assisted PEG-PLA polymeric nanoparticles	U87MG tumor-bearing NOD-SCID mice	Intravenous injection	19 Days (every other day)	2 mg/kg (siRNA)	N/A Inhibited tumor growth (p<0.01 vs control)	[56]
	Cell membranes modified liposomes	MC38 tumor-bearing C57BL/6 mice	Intravenous injection	25 Days (every other day)	PTX (10 mg/kg) -Rg3 (15 mg/kg) - lipo	79.54% tumor inhibition rate (p<0.0001 vs control)	[70]
GOx delivery to accelerate glucose depletion	Biodegradable copper-doped calcium phosphate nanoparticles	4T1 tumor-bearing BALB/c mice	Intratumoral injection	14 Days (Day 1, 5, 9)	10 mg/kg (PGC)	94.9% tumor inhibition rate (p<0.0001 vs control)	[79]
	Liquid metal nanoparticles	CT26 tumor-bearing BALB/c mice	Intravenous injection	25 Days (Day 1, 6 LMGC + laser radiation)	/	N/A Reduced tumor volume (p<0.01 vs LMGC group)	[80]
	Fifth-generation poly(amidoamine) dendrimers	CT26 tumor-bearing BALB/c mice	Intratumoral injection	21 Days (Day 1, 5, 9)	100 μL for each mouse ([Mn]= 50 μg, [GOx] = 250 ng, and [cGAMP] = 5 μg)	99.2% tumor inhibition rate	[81]
	Platelet-derived exosomes	H22 primary tumor-bearing ICR mice	Intravenous injection	14 Days (Day 0, 3, 6, 9, 12, 14 FG@PEL + photothermal therapy)	100 μL 50 mg/mL FG@PEL	N/A Highest median survival rates (p<0.001 vs control)	[82]
Codelivery GOx and oxygen to accelerate glucose depletion	Perfluoropentane nanoparticles	K7M2 osteosarcoma-bearing BALB/c mice	Intravenous injection	22 Days (Day 0, 4, 8, 14, 20 M-mFeP@O_2_-G + NIR)	100 μL 1 mg/mL M-mFeD	90.5% tumor growth inhibition rate (p<0.001 vs control)	[120]
	Hollow bismuth selenide nanoparticles	4T1 tumor-bearing BALB/c mice	Intratumoral injection	21 Days (Day 0, 4, 8, 12 BGPO + laser irradiation)	0.25 mg/kg (GOx)	96.8% tumor growth inhibition rate (p<0.01 vs control)	[88]
	Metal-protein-polyphenol capsules	4T1 tumor-bearing nude mice	Intravenous injection	18 Days (Day 0, 3, 6, 9, 12, 15)	10 mg/kg (ZHOGTA)	N/A Reduced tumor volume (p<0.01 vs control)	[84]
Co-delivery of GOx and oxygen-generating compounds to accelerate glucose depletion	Manganese- and copper-doped carbon dots	4T1 tumor-bearing nude mice	Intravenous injection	14 Days (γ-PGA@GOx@Mn,Cu-CDs NPs + laser irradiation)	100 μL 0.25 mg/mL γ-PGA@GOx@Mn,Cu-CDs NPs	N/A Inhibited tumor growth rate (p<0.001 vs control)	[89]
	Manganese- and copper-doped carbon dots	4T1 xenograft tumor-bearing BALB/c mice	Intravenous injection	14 Days (Day 0, 2, 4)	20 mg/kg (MnDIG@PEG)	N/A Inhibited tumor growth rate (p<0.001 vs control)	[87]
	Platinum nanodendrites	HeLa xenograft tumor-bearing BALB/c mice	Intratumoral injection	28 Days (3 times per week)	15 μg each mouse (NM_Doxo-GOx_)	N/A Significant fold-change in tumor volume (p<0.05 vs Vehicle)	[91]
	Graphitic carbon nitride nanosheets	U87 tumor-bearing Balb/c mice	Intravenous injection	12 Days (Day 0, 2, 4, 6, 8, 10 PCMGH + laser irradiation)	100 μL 200 μg/mL PCMGH	N/A Reduced tumor volume (p<0.001 vs control)	[93]
Hexokinase	Tumor-targeted liposomes	Pan-02 tumor-bearing C57BL/6 mice	Intravenous injection	16 Days (Day 0, 2, 4, 6, 8, 10, 12, 14)	/	N/A Reduced tumor volume (p<0.001 vs control)	[104]
Pyruvate kinase M2	Mesoporous polydopamine nanoparticles	CT26-Luc tumor-bearing Balb/c mice (early liver metastasis model of colorectal cancer)	Intravenous injection	10 Days (Day 1, 3, 5, 7, 9)	5 mg/kg (SHK@HA-MPDA)	N/A Reduced liver nodules (p<0.05 vs SHK group)	[111]
	Prussian blue nanoparticles	HCT116 tumor-bearing Balb/c nude mice	Intravenous injection	21 Days (Day 0, 2, 4 MPB-3BP@CM NPs + laser irradiation)	30 mg/kg (MPB-3BP@CM NPs)	N/A Inhibited tumor growth rate (p<0.0001 vs Control)	[112]
	PKM2 allosteric converter	786-O xenograft tumor bearing Balb/c nude mice	Intravenous injection	28 Days (Day 0, 2, 4, 6, 8)	10 mg/kg (PAC)	N/A Reduced tumor volume (p<0.001 vs control)	[113]
	Multivalent helical polypeptides nanocomposites	MCF-7 tumor-bearing nude mice	Intravenous injection	17 Days (Day 1, 5 (D-I/P@HSA NCs); Day 2, 6 (laser irradiation))	1.25 mg/kg (siRNA) 2.25 mg/kg (ICG)	100% survival rate within 50-days	[114]
	Spherical helical polypeptides nanocomposites	A549/ADR tumor-bearing nude mice	Intravenous injection	21 Days (Day 0, 6)	1.5 mg siRNA/kg and 4 mg DOX/kg	80% survival rate	[115]

Direct cross-study comparisons should be interpreted with caution due to differences in tumor models, dosing regimens, administration routes, and experimental conditions

#### *Limitations and challenges*

While GLUT1 inhibition holds promise for targeting tumor metabolism, it is important to address several clinical concerns related to its therapeutic application. As GLUT1 is highly expressed in erythrocytes, it’s non-selective inhibition would impair glucose uptake in red blood cells, disrupting their energy metabolism and leading to reduced oxygen transport capacity. This may result in anemia, which could pose a substantial risk to patients undergoing GLUT1-targeted therapies. Moreover, the inhibition of GLUT1 expression in the BBB could compromise the glucose supply to the brain, potentially leading to neurotoxic effects. This is particularly relevant for brain tumors or therapies involving systemic drug delivery to brain tissue, as the disruption of glucose homeostasis in the brain could lead to cognitive deficits or other neurological side effects.

Although nanomedicine-based delivery of 2-DG could significantly reduce its systemic exposure and lower off-target neurotoxicity by improving tumor accumulation and controlled release, current nanomedicines failed to completely eliminate the risk of neurotoxicity, especially at higher doses or during repeated administration. These limitations and mitigation strategies have been critically discussed in the section on glycolysis inhibition and were added to emphasize the translational challenges of 2-DG-based metabolic therapy.

### Nanomedicine designed to accelerate glucose depletion via GOx-based starvation therapy

Tumor cells typically exist in an energetically demanding state due to their rapid proliferation requirements. Although glycolysis provides a rapid energy supply, it exhibits low glucose utilization efficiency, rendering tumor cells exquisitely sensitive to glucose metabolism perturbations [71]. Consequently, accelerating glucose depletion has emerged as a novel metabolic therapeutic strategy for cancer. Its core mechanism involves actively depleting glucose within the tumor microenvironment (TME), thereby disrupting the dysfunctional Warburg effect that tumor cells rely upon while simultaneously remodeling the immune microenvironment. Given that tumor cells consume glucose at a rate 20–30 times higher than normal cells, accelerating their glucose consumption directly induces apoptosis via energy deprivation [72, 73]. This is compounded by reduced nicotinamide adenine dinucleotide phosphate (NADPH) production, compromising antioxidant capacity and leading to ROS accumulation that damages mitochondria [74]. Furthermore, accelerated glucose depletion alleviates T cell suppression and promotes the repolarization of immunosuppressive M2 macrophages towards antitumor M1 phenotypes, ultimately reversing tumor immunosuppression [75, 76]. Strategies for accelerating glucose depletion in tumor cells mainly include the delivery of glucose oxidase (GOx), co-delivery of GOx and oxygen, co-delivery of GOx and oxygen-generating compounds, and photodynamic/photothermal-driven glucose depletion. And, residual oxygen (even at < 1% O₂) in tumors can trigger minimal GOx activity, catalyzing the initial conversion of glucose to gluconic acid and H₂O₂. This small, initial production of H₂O₂ creates a local increase in oxygen concentration through reactions with catalytic nanozymes, which further converts H₂O₂ into oxygen. This oxygen is then used to support the self-amplifying catalytic cycle, providing enough oxygen for the GOx to continue depleting glucose, maintaining the metabolic blockade. In the following text, a detailed introduction will be provided on how nanomedicines can facilitate these strategies to accelerate glucose depletion in tumor cells and combat tumors Table 2.

**Table 2:** A summary of combinatorial therapeutic strategies targeting glucose metabolism for cancer treatment

**Strategies**	**Carrier**	**Therapeutic agent**	**Advantages**	**Disadvantages**	**Ref**
Glucose metabolic regulation + chemotherapy	Self-assembled nano-PROTACs constructed from PROTAC and DOX	DOX	• Enhanced immune therapy effect • Increased drug stability	• Technical complexity • Potential immune adverse events	[127]
Glucose metabolic regulation + radiotherapy	Fluorinated calcium carbonate nanocomposite	PFCE	• Enhanced radiotherapy efficacy • Targeted delivery • Reduced side effects	• Potential long-term effects • Complexity in clinical application	[128]
Glucose metabolic regulation + immunotherapy (anti PD-L1)	HA coated hybrid nanoparticles constructed from GOx and manganese ions	GOx	• Enhanced anti-tumor immunotherapy • Superior tumor targetability	• Treatment tolerance issues • Potential immune adverse events	[133]
	Lung cancer cell membranes camouflaged Cu-substituted layered double hydroxide nanoparticles	GOx	• Enhanced anti-tumor immunotherapy • Targeted delivery and reduced side effects	• Complexity in formulation (multi-step encapsulation process and pH-sensitive linker stability) • Potential for drug resistance	[134]
	Mn-based galvanic cell	/	• Bidirectional synergistic therapy • Superior tumor targetability	• Technical complexity and cost • Potential immune adverse events	[135]
Glucose metabolic regulation + immunotherapy (anti CTLA-4)	Chondroitin sulfate-modified zeolitic imidazolate framework-8	2-DG, BAY-876, and chloroquine	• Alleviated immunosuppression • Superior tumor targetability	• Individual differences • Complexity in formulation (multi-step manufacturing process and membrane coating uniformity)	[136]
Glucose metabolic regulation + chemoimmunotherapy	Poly(amino acid)	OXA-ASP prodrug	• Enhanced chemoimmunotherapy effect • Reduced chemotherapy resistance • Precise targeting and delivery	• Complexity of drug interactions • Individual differences • Potential immune toxicity	[137]
Glucose metabolic regulation + PCD	Tannic acid coordinated vanadium oxides camouflaging with PD-L1 inhibitory peptides modified tumor cell membranes	LND	• Enhanced cancer cell death • Superior tumor targetability • Activated anti-tumor immune response	• Complexity of drug interactions • Potential immune adverse events	[144]
	SH-PEG-Da encapsulated OMVs	Fe ions and Au NPs	• High safety • Precise drug delivery • Synergistic therapeutic effect	• Production and quality control • Limited drug loading capacity	[145]
	FeOOH nanoshuttles	Au nanodots and iron-Ap complexes	• Reduced tumor resistance • Synergistic effect of dual cell death pathways	• Potential toxicity v Complexity of treatment • Individual differences	[147]
	HA coated Cu-based nanoneedles	GOx and BSO	• Synergistic effect of dual cell death pathways • Superior tumor targetability	• Complexity in clinical application • Potential immune adverse events	[149]
Glucose metabolic regulation + OXPHOS	Glycopolymer containing a caged H_2_S/H_2_O_2_ dual-donor	GOx	• Dual metabolic intervention • Enhanced immune response	• Potential metabolic disorders • Challenges in monitoring and management	[155]
	Manganese ferrite nanoparticles	DCA	• Enhanced immune response • Precise targeting • Improved tumor microenvironment	• Potential immune toxicity • Complexity of drug interactions • Unclear biodistribution and clearance	[156]
Glucose metabolic regulation + glutamine metabolic regulation	Detachable copolymer shell and a ROS-sensitive degradable MOF core	GOx and BPTES	• Reversal of metabolic abnormalities • Dual-starvation therapy • Superior tumor targetability	• Potential metabolic disorders • Complexity of treatment • Potential toxicity of the nanoreactor	[160]
Glucose metabolic regulation + PTT	OMV	GOx and OA	• Oxygen-regulating function • Precise tumor targeting	• Potential immune response • Difficulty in precise control of drug release	[25]
	Ce-Mn heterojunction	GOx	• Cancer-specific enzymatic activity • Good biocompatibility and degradability	• Complex synthesis and preparation process • Potential immunogenicity • Uncertain long-term efficacy	[165]
	Polyvinylpyrrolidone	My and iron ions	• Precise targeting • Safety of low-temperature PTT • Anti-metastatic inflammatory process	• Complex synthesis and preparation process • Potential immune and toxicological responses	[168]
Glucose metabolic regulation + PDT	Targeting peptides modified photosensitizer chlorin e6 conjugating with lecithin	siGLUT1	• Self-amplifying energy-depleting mechanism • Good biocompatibility and degradability	• Potential immune adverse events • Individual differences • Uncertain long-term efficacy	[170]
	mSiO₂ shell encapsulating LnNPs	2-DG and Ce6	• Advantages of precise imaging guidance • Innovative metabolic interference	• Complexity of the therapeutic regimen design • Potential immune responses	[171]
Glucose metabolic regulation + matrix modulation	Cationic liposomes	TPCA-1 and AsiG	• Dual metabolic rectification • Precise tumor targeting • Enhanced immune response	• Complex preparation process • Long-term efficacy and drug resistance issues	[180]
	Mesothelin-targeted nanoparticles	CXCL1 siRNA and WT	• Improved tumor microenvironment • Precise tumor targeting	• Limited drug delivery efficiency • Potential drug interactions	[184]

#### Direct GOx delivery systems

As an ideal endogenous oxido-reductase, GOx catalyzes the oxidation of glucose, consuming oxygen to produce gluconic acid and hydrogen peroxide (H₂O₂), with a single GOx molecule consuming approximately 1.2 × 10⁴ glucose molecules per minute. Beyond its rapid and efficient glucose depletion capacity, GOx simultaneously induces tumor cell death by generating H₂O₂, which triggers oxidative stress and causes DNA damage [77]. However, as a protein therapeutic, GOx is inherently susceptible to denaturation and exhibits an extremely short plasma half-life, coupled with potential off-target toxicity [78]. Furthermore, its application is fundamentally constrained by a critical paradox: while GOx requires oxygen for its catalytic activity, the tumor microenvironment is typically hypoxic. Therefore, leveraging nanotechnology to enhance GOx stability and achieve tumor-targeted delivery is paramount for improving its therapeutic efficacy. Furthermore, strategically co-delivering oxygen or oxygen-generating substances to address its oxygen dependency during glucose catalysis is also critical for successful treatment.

To address the challenge of intra-tumoral delivery of GOx, Huang et al. developed PGC-DOX nanomedicines using PEG-modified GOx as a template [79]. Through a one-step biomineralization approach, biodegradable copper-doped calcium phosphate (CuCaP) nanoparticles were formed to load doxorubicin (DOX). GOx catalyzed glucose oxidation to generate H₂O₂, not only depleting tumor energy for starvation therapy but also enhancing chemodynamic therapy. Combined with DOX-based chemotherapy, PGC-DOX created a multimodal anti-tumor effect. In 4T1 tumor-bearing mice, intratumoral injection of PGC-DOX achieved a 94.9% tumor inhibition rate, while intravenous injection resulted in a 77.8% inhibition rate, which is both significantly higher than control groups.

**Fig. 4:**
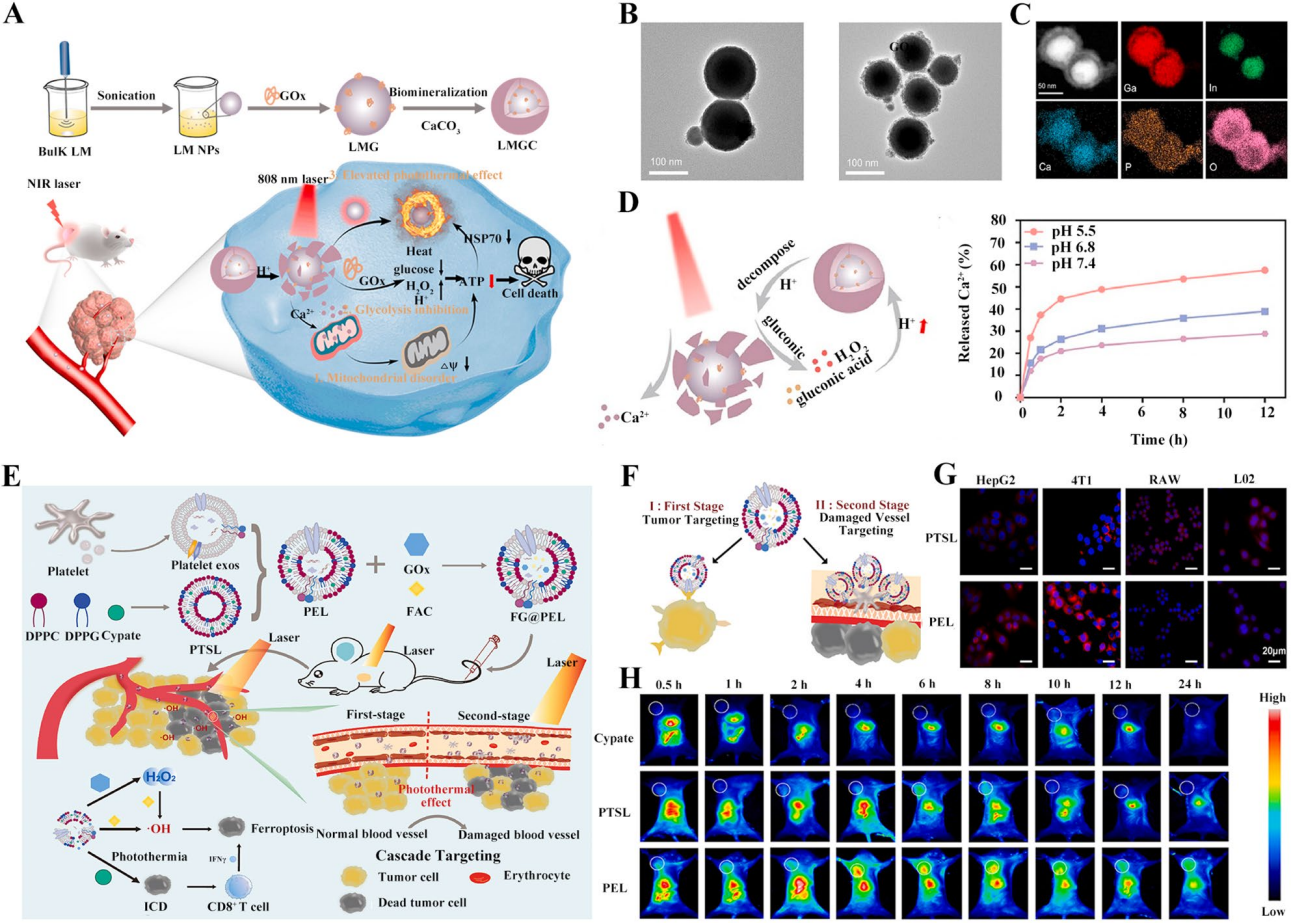
Nanomedicine for direct GOx delivery. (**A**) Schematic diagram of LMGC design, preparation, in vivo transport, and action mechanism. (**B**) LMGC exhibited a spherical-like morphology, and (**C**) the distribution diagram of various elements on the nanomedicine was as presented. (**D**) Acid-triggered drug release profiles of LMGC [80]. Copyright © 2022 Elsevier. (**E**) Schematic diagram of FG@PEL design, preparation, in vivo transport, and action mechanism. (**F**) Schematic diagram of targeting mechanism of FG@PEL. (**G**) FG@PEL could selectively target tumor cells and (**H**) accumulated at tumor region in tumor-bearing mice [82]. Copyright © 2022 Elsevier

Biomineralization refers to the phenomenon where living organisms form minerals in specific sites through their own physiological processes, calcium ions are excellent mediators for biomineralization. Moreover, liquid metal (LM) has emerged as a novel drug delivery vector due to its high biocompatibility, flexible surface functionalization, excellent photothermal properties, and responsiveness to the tumor microenvironment, particularly demonstrating efficient loading capacity for substances such as enzymes. Hence, Zhang et al. combined the protein delivery potential of LM with the biomineralization capability of calcium ions. They designed a nanomedicine with a photothermal-convertible LM core loaded with GOx, which was then encapsulated by a calcium carbonate (CaCO₃) mineralization layer and surface-modified with polyethylene glycol-polyaspartic acid (PEG-PAsp) to enhance water solubility and stability (Fig. 4A). The resulting nanomedicine LMGC exhibited homogeneous quasi-spherical morphology with negligible aggregation (Fig. 4B). As shown in Fig. 4C, elemental mapping distinctly visualized the spatial distribution of constituent elements within the nanostructure. The LMGC exhibited pH-triggered degradation and burst drug-release capabilities. As presented in Fig. 4D, at pH 5.5, the CaCO₃ layer decomposed to release Ca²⁺ and GOx, which depleted glucose to starve tumor cells [80].

Owing to their structural controllability, versatile modifiability, and stimuli-responsive cargo release properties, dendrimers serve as ideal carriers for protein drug delivery. Consequently, Shi et al. synthesized fifth-generation poly(amidoamine) (G5 PAMAM) dendrimers. These were surface-modified with methoxy poly(ethylene glycol) (mPEG) and phenylboronic acid, encapsulating 2.8 nm MnO₂ nanoparticles to form MGPP nanoparticles (MGPP NPs). GOx was subsequently loaded via physical adsorption, while cyclic GMP-AMP (cGAMP) was incorporated through electrostatic interactions, achieving encapsulation efficiencies exceeding 98% for both agents [81].

Platelet-derived exosomes are nanoscale vesicles secreted by platelets, possessing innate tumor-targeting ability, drug-loading capacity, and enrichment capability in damaged blood vessels. Based on these properties (Fig. 4E), Li et al. developed a light-controlled nanomedicine (FG@PEL) by fusing platelet exosomes with photothermal-sensitive liposomes (PTSL) mediated by PEG8000, loading GOx and ferric ammonium citrate [82]. Platelet exosomes actively targeted tumor cells through P-selectin-CD44 receptor binding. After photothermal effect-induced vascular damage, the nanomedicine further recruited nanoparticles for cascade-responsive enrichment in tumor sites (Fig. 4F). Due to the photothermal effect at 45℃, both the H₂O₂ production by GOx and the ·OH generation efficiency catalyzed by FAC were significantly higher than those at 37℃. As shown in Fig. 4G and H, both in vivo and in vitro targeting experiments revealed that FG@PEL could achieve immune evasion and target-specific accumulation in tumor cells. In a 4T1 breast cancer lung metastasis model, the FG@PEL + laser group exhibited a 70% reduction in the number of lung metastatic nodules and a significant decrease in the Ki-67 proliferation index.

Although GOx activity is constrained in severely hypoxic tumor regions due to the lack of available oxygen, a bootstrap mechanism allows for minimal GOx activity even at < 1% O₂. In this low-oxygen environment, residual oxygen can catalyze the initial conversion of glucose to gluconic acid and hydrogen peroxide (H₂O₂). This small production of H₂O₂ is then converted into oxygen by catalytic nanozymes (e.g., PdPt-based nanozymes), providing enough oxygen to sustain a self-amplifying cycle that continues glucose depletion and oxidative stress. Although GOx has shown promise in targeting tumor metabolism, its immunogenicity is a concern. GOx is a foreign protein that can trigger immune responses, including antibody production and inflammatory reactions, which may lead to reduced efficacy over time. To mitigate these immune responses, there might be several strategies, including PEGylation, nanomedicines for GOx encapsulation, site-specific modifications, etc.

#### Co-delivery with oxygen carriers

**Fig. 5:**
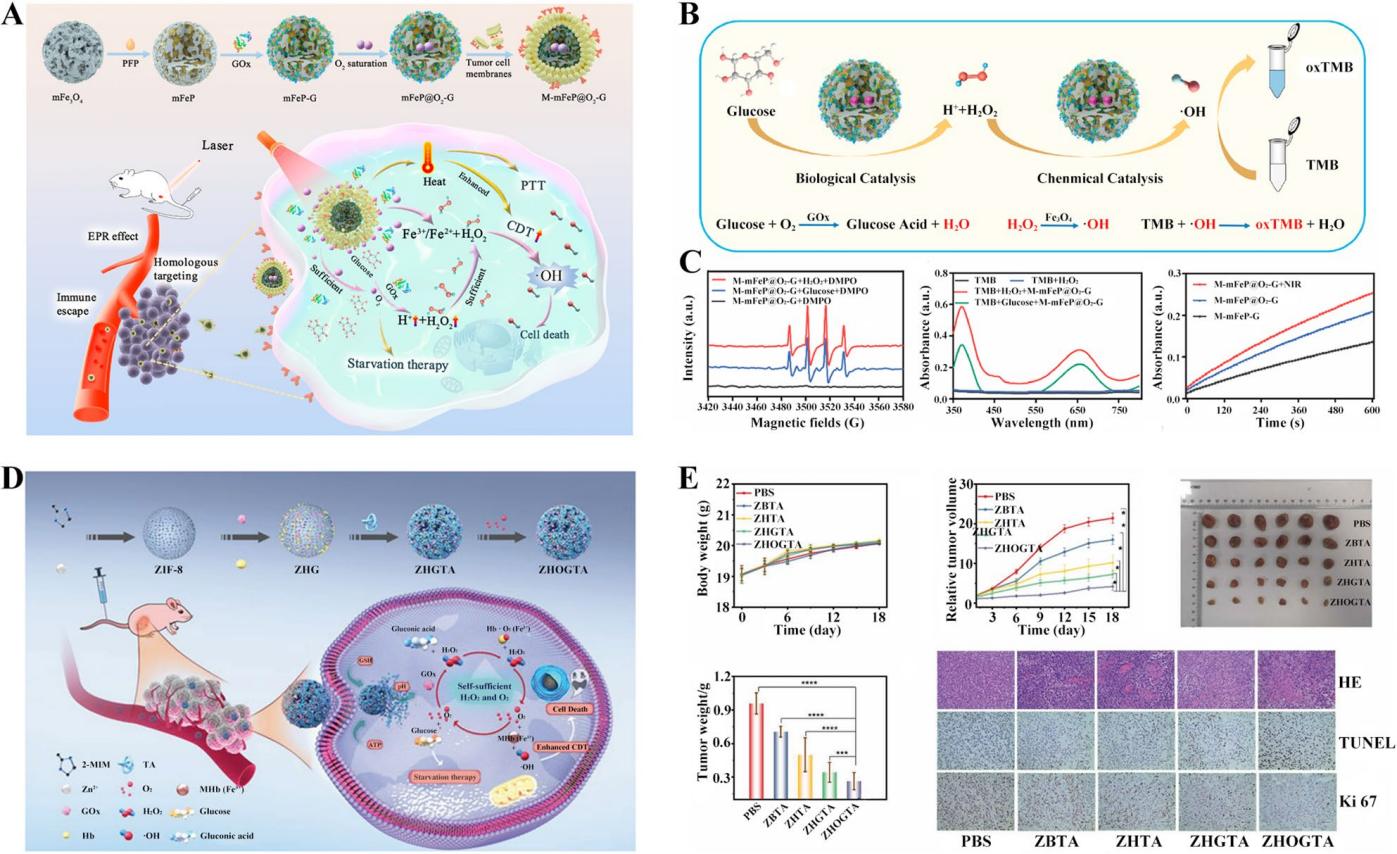
Nanomedicine for co-delivery of GOx and oxygen. (**A**) Schematic diagram of M-mFeP@O₂-G design, preparation, in vivo transport, and action mechanism. (**B**) The catalytic mechanism of M-mFeP@O₂-G. (**C**) The catalytic performance of M-mFeP@O₂-G in vitro [87]. Copyright © 2023 Elsevier. (**D**) Schematic diagram of ZHOGTA design, preparation, in vivo transport, and action mechanism. (**E**) Antitumor efficacy and safety assessment of ZHOGTA on 4T1-tumor bearing mice, including changes of body weight, tumor volume measurements, tumor imaging, and histopathological analysis of excised tumors. *p < 0.05 [84]. Copyright © 2022 Wiley

The catalytic process of glucose consumption by GOx requires oxygen, but this is constrained by the hypoxic tumor microenvironment [83, 84]. Therefore, to enhance the anti-tumor efficacy of glucose depletion via GOx, it is promising to develop GOx and oxygen (O₂) co-delivery strategies. Ascribed to the presence of highly electronegative fluorine atoms within its structure, perfluoropentane (PFP) can form stable interactions with O₂ via van der Waals forces [85, 86]. This imparts a high O₂ solubility to PFP, approximately 20-fold higher than that of water, thus demonstrating its significant O₂-carrying capacity. Building on the O₂-carrying capacity of PFP, Li et al. constructed M-mFeP@O₂-G nanoparticles by employing mesoporous Fe₃O₄ (mFe₃O₄) as the core [87]. As shown in Fig. 5A, this core was sequentially functionalized by loading PFP for oxygen carriage and GOx for glucose depletion, followed by encapsulation with a tumor cell membrane to confer tumor-targeting capability. As illustrated in the catalytic mechanism of M-mFeP@O₂-G (Fig. 5B), GOx catalyzed glucose to generate H₂O₂, which subsequently underwent a Fenton reaction mediated by mFe₃O₄ to enhance tumor cell killing. Concurrently, PFP-loaded oxygen accelerated GOx-catalyzed glucose oxidation, establishing a self-amplifying therapeutic cycle. As shown in Fig. 5C, the M-mFeP@O₂-G possessed time-dependent enhancement of catalytic performance with significantly higher ROS production observed at increased glucose concentrations. Under sufficient oxygen supply, GOx-mediated glucose depletion was significantly enhanced, thereby starving tumor cells and achieving a tumor inhibition rate of 90.5% in a subcutaneous K7M2 osteosarcoma model.

Moreover, Xue et al. engineered a multifunctional nanoreactor using hollow bismuth selenide (Bi₂Se₃) nanoparticles as the core framework [88]. Then, GOx electrostatically adsorbed onto the Bi₂Se₃ surface via polyethyleneimine (PEI) coating; while oxygen-loaded PFC encapsulated within the Bi₂Se₃ cavity, forming the nanoreactor (BGPO NPs). By leveraging the photothermal conversion effect of Bi₂Se₃, BGPO NPs released oxygen under NIR light irradiation, significantly increasing dissolved oxygen concentration to alleviate tumor hypoxia. The released oxygen promoted GOx-mediated glucose consumption and inhibited ATP production. As a result, BGPO NPs adopted a synergistic strategy of oxygen self-supplying starvation therapy and sensitized photothermal therapy, effectively overcoming tumor hypoxia and thermotolerance. The tumor growth inhibition (TGI) rate reached 96.8%, tumor volume decreased by over 80% compared to the control group, and the survival period was prolonged by more than 42 days.

As an oxygen-transport protein specifically expressed in erythrocytes, hemoglobin (Hb) reversibly coordinates molecular oxygen via ferrous ions within its heme groups. This tetrameric metalloprotein serves as the physiological carrier for systemic oxygen delivery. However, due to the inherent structural vulnerability to conformational denaturation and proteolytic degradation in complex physiological environments, coupled with inefficient tumor-specific accumulation, therapeutic utility of Hb in oncology applications was substantially limited. Hence, Xu et al. developed metal-protein-polyphenol capsules through templated assembly on zeolitic imidazolate framework-8 (ZIF-8), co-immobilizing GOx and Hb within a polyphenol network matrix. The differential oxygen affinity between Hb and methemoglobin enabled spatiotemporally controlled oxygen release during glycolysis. As shown in Fig. 5D, following cellular internalization, the obtained nanomedicine ZHOGTA released GOx to deplete intratumoral glucose while concurrently liberating oxygen to mitigate hypoxia-induced suppression of GOx catalytic activity. In 4T1 tumor-bearing mice treated with ZHOGTA via tail vein injection over an 18-day period, tumor growth was significantly suppressed compared to control groups, while body weight remained stable, demonstrating favorable therapeutic efficacy and safety (Fig. 5E). This self-replenishing oxygen cascade compensated for enzymatic oxygen consumption, establishing an oxygen-autonomous cycle that potentiated glycolytic flux and enhanced glucose-deprivation therapy efficacy [84].

For the initial activation of GOx, exogenous oxygen carriers such as PFP or hemoglobin are often not just an optional addition but an essential strategy for triggering GOx activity, especially in tumors with significantly low baseline oxygen levels. These carriers can deliver and release oxygen locally, providing the necessary oxygen supply to activate GOx and sustain its catalytic cycle in the initial stages.

#### Co-delivery with oxygen-generating nanozymes

GOx consumes both oxygen and glucose to produce H₂O₂, which can subsequently serve as a precursor for oxygen generation. Therefore, the rational enzymatic catalysis of H₂O₂ to regenerate oxygen offers a strategy to provide O₂, thereby enhancing the efficacy of GOx-mediated glucose depletion. For this purpose, Sun et al. employed manganese- and copper-doped carbon dots (Mn, Cu-CDs; denoted as Cu-CDs) as self-sustaining oxygenator nanodots to catalyze hydrogen peroxide decomposition for oxygen generation [89, 90]. For preparing the nanomedicine, core-shell nanoparticles were fabricated through electrostatic interactions or mild chemical crosslinking between GOx-modified Cu-CDs and γ-polyglutamic acid (γ-PGA). The nanoparticles leveraged γ-glutamyl transpeptidase (GGT)-mediated endocytosis to target tumor cells, enhancing tumoral accumulation. Within the tumor site, the released GOx catalyzes glucose oxidation to gluconic acid and H₂O₂, depleting glucose in the tumor microenvironment to induce starvation therapy. Concurrently, Cu-CDs facilitated H₂O₂ decomposition into O₂, alleviating tumor hypoxia and potentiating the starvation therapy efficacy. In terms of therapeutic efficacy, when compared to the control group, the GOx-loaded nanoparticles depleted glucose and reduced intracellular ATP levels by 60% (compared to the control group). Meanwhile, Cu-CDs catalyzed the generation of O₂ from H₂O₂, increasing intratumoral oxygen content by 1.83-fold.

Manganous-manganic oxide (Mn₃O₄) demonstrates unique advantages in tumor therapy through its catalytic generation of O₂ from H₂O₂, exhibiting strong pH adaptability and high synergistic immunostimulatory effects. However, its performance requires further optimization regarding deep tumor penetration, manganese ion toxicity control, and multi-component release regulation. To amplify the therapeutic effect of GOx by leveraging the oxygen-producing catalytic ability of Mn₃O₄, Lin et al. constructed disulfide-bonded dendritic mesoporous organosilica nanoparticles (DMONs). These nanoparticles were surface-modified with Mn₃O₄ and loaded with the IDO inhibitor Epacadostat (IDOi) and GOx through the pore channels of DMONs, and further surface-modified with PEG to prepare a TME-responsive multifunctional nanocomposite. In the weakly acidic (pH 5.5) and high-glutathione (GSH)-concentration TME, the disulfide bonds in DMONs break, causing rapid degradation of the nanocomposite and releasing Mn²⁺, IDOi, and GOx. Mn₃O₄ decomposed H₂O₂ to generate O₂, relieving tumor hypoxia to promote GOx-mediated glucose consumption for improved starvation therapy [87].

**Fig. 6:**
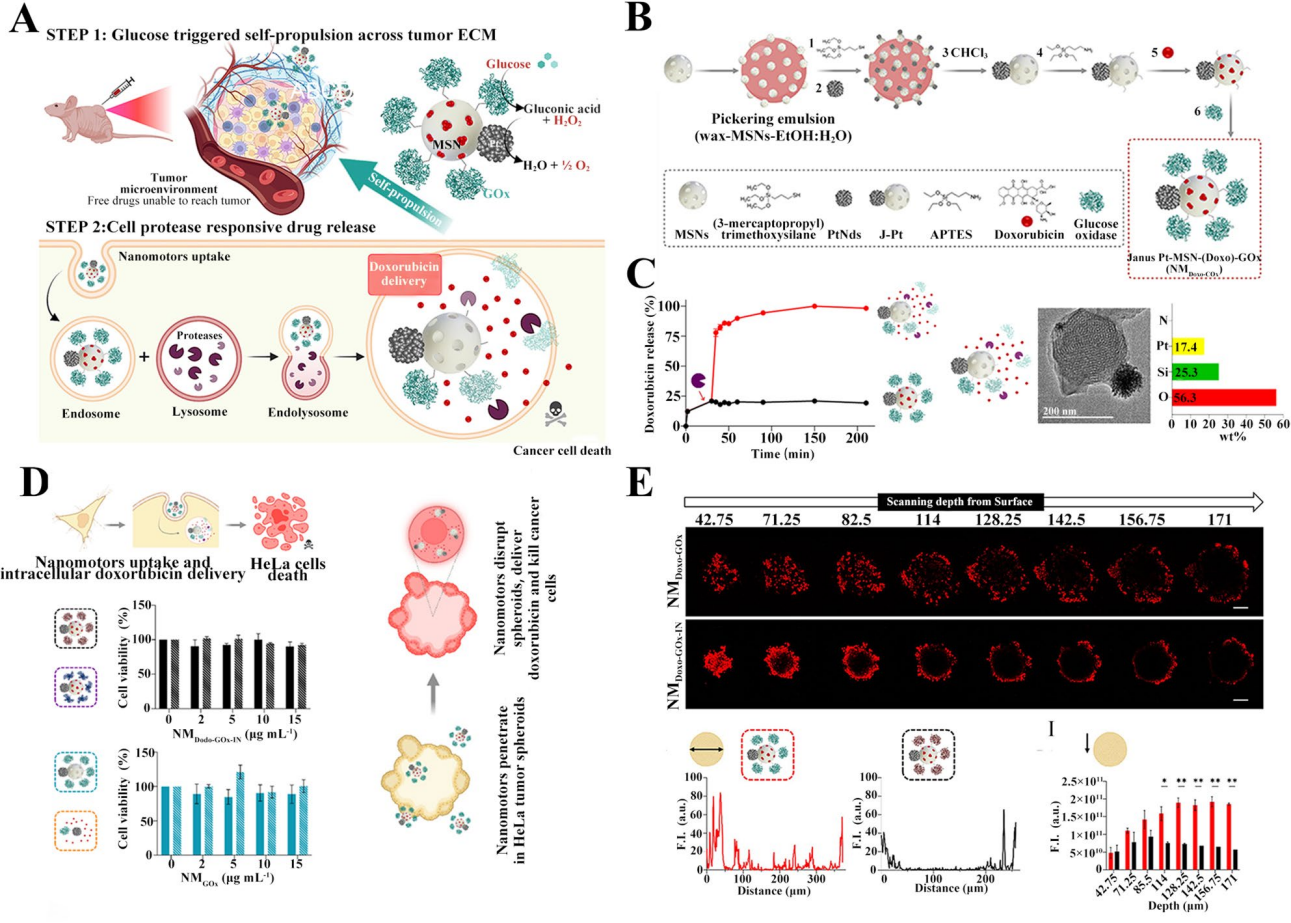
Nanomedicine for co-delivery of GOx and oxygen-generating compounds. (**A**) Schematic diagram of nanomotor design, in vivo transport, and action mechanism. (**B**) The preparation process of nanomotor. (**C**) The morphology, zeta potential, and burst-release drug release behavior of nanomotor. (**D**) Antitumor mechanism and efficacy of nanomotor towards HeLa cells. (**E**) Deep tumor penetration behavior of nanomotor in HeLa tumor spheroids [91]. Copyright © 2025 American Chemical Society

As an alternative enzymatic oxygen generator, platinum nanodendrites (PtNds) functionally mimic peroxidase to catalyze oxygen from hydrogen peroxide. Concurrently addressing intratumoral penetration barriers, Ramón Martínez-Máñez and colleagues engineered glucose-driven Janus nanomotors for solid tumor therapy [91]. The well-designed glucose-fueled gated nanomotors established a self-sustaining therapeutic-diagnostic cascade: (i) GOx consumed glucose to initiate starvation while generating H₂O₂; (ii) PtNds converted H₂O₂ into O₂ and propulsion energy; (iii) O₂ could help GOx better consume glucose to form a positive feedback, while autonomous motion overcame interstitial pressure barriers (Fig. 6A). For preparation in Fig. 6B, the nanomotor was constructed using mesoporous silica nanoparticles (MSNs) as the core framework through a sequential process: surface silanization, doxorubicin (DOX) loading within mesopores, and surface anchoring of GOx. In vitro potential change measurements validated the stepwise assembly of the nanomotors (Fig. 6C). Concurrently, DOX release profiles demonstrated protease-triggered on-demand drug release, effectively preventing nonspecific leakage observed in conventional chemotherapy. In simulated extracellular matrix (ECM) and tumor spheroid models, glucose-fueled gated nanomotor movement endowed them with enhanced penetration ability, enabling deep drug delivery into tumor tissues and reducing cell viability (Fig. 6D and E). Furthermore, the study evaluated the therapeutic efficacy of nanomotors using both early- and late-stage HeLa cell-derived subcutaneous xenograft models. In the early-stage model which treatment was initiated at tumor volume ≈ 25 mm³, nanomotors significantly suppressed tumor growth, with treated tumors reaching only one-third the volume of controls. Remarkably, in the advanced-stage model, the nanomotors maintained significant tumor growth retardation even during progressive disease, whereas non-motile control particles showed comparable tumor volumes to untreated groups. These findings demonstrated that this nanomotor-driven delivery strategy overcame critical drug penetration barriers in established solid tumors, offering substantial therapeutic advantages. The PGI NPs constructed by Xue et al. formed a synergistic antitumor system by integrating nanozyme-catalyzed oxygen production, sonodynamic therapy, and metabolic exhaustion strategies [92]. PdPt nanozyme was first synthesized, followed by surface modification with SH-PEG-NH₂. Glucose oxidase (GOx) was covalently conjugated to the surface, and the sonosensitizer IR780 was subsequently loaded through electrostatic interactions to obtain a bimetallic PdPt-based nanocatalyst, designated as PdPt@GOx/IR780 (PGI). It disrupted the glucose metabolism dependence of tumors through a cascade reaction of glucose consumption-hydrogen peroxide generation-oxygen regeneration, while simultaneously alleviating hypoxia and enabling GOx-mediated glucose starvation therapy with self-supplied oxygen to amplify therapeutic efficacy. In the 4T1 tumor-bearing mice, PGI achieveed efficient tumor suppression through the synergistic effects of starvation therapy, photothermal therapy (PTT), and sonodynamic therapy (SDT), with favorable biosafety.

Graphitic carbon nitride (C₃N₄), a metal-free semiconductor featuring a distinctive graphene-like layered topology, exhibits photocatalytic activity for oxygen evolution via water splitting. Hence, Yin et al. engineered a nanoplatform using PDA nanospheres as a substrate. These were integrated with C₃N₄ nanosheets and enveloped with MIL-100 (Fe) metal-organic frameworks, followed by GOx loading and HA surface functionalization for tumor targeting. This nanomedicine enabled self-sufficient oxygen production through catalytic water splitting to modulate the hypoxic tumor microenvironment for enhanced GOx-mediated glucose consumption [93]. In U87 glioblastoma-bearing Balb/c mice, the nanomedicine administration combined with dual-laser irradiation achieved over 90% tumor growth suppression, with no appreciable systemic toxicity observed in major organs as confirmed by histological analysis.

#### Integration with physical therapies

Beyond direct oxygen delivery or oxygen-generating agents, researchers have innovatively improved tumor oxygenation by normalizing aberrant vasculature, thereby potentiating glucose oxidase-mediated glucose depletion. Additionally, priming strategies such as tumor vasculature normalization or photothermal therapy (PTT)-induced transient oxygenation can temporarily improve oxygen availability in the tumor, enabling GOx activation. Innovatively leveraging photothermal effects to normalize tumor vasculature for enhanced intratumoral oxygen supply, Xie et al. co-assembled GOx, indocyanine green (IR820), and α-cyano-4-hydroxycinnamic acid (CHC) into nanoparticles (IGC/NPs) [93]. In this nanosystem, IR820 could mediate photothermal conversion to disrupt tumor vasculature, GOx could deplete glucose, while CHC could inhibit monocarboxylate transporter 1 to block lactate uptake. This study established a CT26 tumor-bearing mouse model (subcutaneous xenografts), liver/lung metastasis mouse model (intrasplenic/caudal vein injection of CT26 cells), and post-surgical recurrence mouse model (observing recurrence after CT26 tumor resection) to evaluate the therapeutic efficacy of nanomedicines. Results showed that GC/NPs combined with NIR light irradiation achieved a tumor volume inhibition rate of over 80%, significantly outperforming monotherapy. In liver/lung metastasis models, the IGC/NPs group exhibited approximately 60% reduction in metastatic foci and 30% prolonged survival time. In the post-surgical recurrence model, the tumor recurrence rate in the IGC/NPs group was 75% lower than the control group. These findings fully validate that the “energy depletion + thermal damage” anti-tumor strategy was achieved by integrating photothermal-enhanced oxygen supply, GOx-mediated glucose consumption, and CHC-induced energy substrate reduction. This novel strategy provides a new paradigm for combined therapy via energy metabolism regulation.

#### Limitations and challenges

Although GOx activity is constrained in severely hypoxic tumor regions due to the lack of available oxygen, a bootstrap mechanism allows for minimal GOx activity even at < 1% O₂. In this low-oxygen environment, residual oxygen can catalyze the initial conversion of glucose to gluconic acid and hydrogen peroxide (H₂O₂). This small production of H₂O₂ is then converted into oxygen by catalytic nanozymes (e.g., PdPt-based nanozymes), providing enough oxygen to sustain a self-amplifying cycle that continues glucose depletion and oxidative stress. Although GOx has shown promise in targeting tumor metabolism, its immunogenicity is a concern. In addition, GOx is a foreign protein that can trigger immune responses, including antibody production and inflammatory reactions, which may lead to reduced efficacy over time. To mitigate these immune responses, there might be several strategies, including PEGylation, nanomedicines for GOx encapsulation, site-specific modifications, and etc. While GOx has shown promise in combination therapies, its efficacy as a monotherapy in severely hypoxic tumor regions remains limited. Several studies have reported reduced efficacy of GOx in such regions, emphasizing the need for supporting strategies to enhance its activity.

### Nanomedicine designed for the modulation of glycolytic enzymes

Tumor cells exhibit high dependency on glycolysis for energy production and biosynthetic precursors, establishing this pathway as a critical antitumor target [94]. Modulating key glycolytic enzymes represents a pivotal therapeutic strategy. Hence, we focus on essential enzymes within this process, their regulatory mechanisms, and how to use nanomedicine for improved enzyme regulation. Hexokinase (HK) catalyzes the initial phosphorylation of glucose to glucose-6-phosphate, thereby facilitating intracellular glucose retention [95]. In tumor cells, the HK2 isoform is frequently overexpressed, promoting glucose uptake and glycolytic flux to support rapid neoplastic proliferation [96]. Phosphofructokinase-1 (PFK-1) catalyzes the second committed rate-limiting step of glycolysis, phosphorylating fructose-6-phosphate (F6P) to fructose-1,6-bisphosphate [97]. In tumor cells, PFK-1 activity is fine-tuned by multiple allosteric modifiers, which are notably activated by adenosine monophosphate (AMP)/adenosine diphosphate (ADP) and inhibited by ATP/citrate [98]. Hence, the regulation of PFK-1 has been considered as one of the effective strategies for anti-tumor therapy [99]. Moreover, PKM2 catalyzes the final step of glycolysis, converting phosphoenolpyruvate (PEP) into pyruvate and generating ATP [100]. As a key regulator of glycolytic flux, PKM2 balances energy production with biosynthetic demands and directly participates in tumor signaling pathways. Lactate dehydrogenase A (LDHA) sustains continuous glycolysis under hypoxic conditions or during the Warburg effect by reducing pyruvate to lactate while oxidizing NADH to regenerate NAD+ [101]. Overexpressed in multiple tumor types, it serves as the pivotal enzyme responsible for massive lactate production in cancer cells. Collectively, rational modulation of these key enzyme activities can block the glycolytic pathway utilized by tumor cells for glucose metabolism, thereby achieving tumor starvation therapy.

#### Nanomedicine for the modulation of hexokinase

Hexokinase (HK) comprises four isoforms from HK1 to HK4 that exhibit tissue-specific distribution patterns [102]. In tumor cells, HK2 is frequently overexpressed across diverse malignancies and is intimately linked to oncogenesis and progression, while HK1 expression is also elevated [103]. Consequently, the modulation of HK1 and HK2 represents strategic approaches for targeted disruption of tumor energy supply.

For example, 3-Bromopyruvate (3-BP) controls tumor glucose metabolism by irreversibly inhibiting HK2 activity via covalent binding. However, its clinical translation is limited by severe adverse effects (e.g., hepatotoxicity), attributable to nonspecific biodistribution, low bioavailability, and a narrow therapeutic window. To solve this problem, Li et al. developed tumor-targeted liposomes (T-Lipo-3BP) to achieve specific delivery of 3-BP. Structurally, T-Lipo-3BP featured 3-BP loaded within the aqueous core of liposomes, with surface-anchored CREKA peptides that specifically recognize fibrin-microthrombi on tumor vascular endothelium. By specifically delivering 3-BP to tumor cells, it effectively inhibited HK1 activity, blocked ATP production in the glycolytic process, and starved tumor cells. In a Pan-02 tumor-bearing C57BL/6 mouse, the intratumoral ATP levels in the T-Lipo-3BP group were significantly lower than those in the free 3-BP group, the tumor volume decreased by approximately 50%, and there was no hepatotoxicity caused by non-specific distribution [104].

Moreover, given that 3-BP can inhibit HK2 but lacks mitochondrial targeting capability with resulted in systemic toxicity and nonspecific effects. Shanta Dhar et al. developed a nanomedicine by loading 3-BP onto triphenylphosphonium (TPP)-functionalized gold nanoparticles (AuNPs) to exploit TPP’s intrinsic mitochondrial homing capability. The obtained nanoparticles exhibited a diameter of 20–30 nm with positive surface charge, enabling efficient targeting of negatively charged mitochondria, where they specifically localize to mitochondrial membrane-bound HK2. And, T-3-BP-AuNP significantly reduced intracellular and extracellular lactate levels (inhibition rate > 80%), suppressed extracellular acidification rate (ECAR), and blocked glucose metabolism through glycolysis [105].

#### Nanomedicine for the modulation of phosphofructokinase-1

PFK-1 is the second rate-limiting enzyme in the process of glycolysis, playing a role in phosphorylating fructose 6-phosphate (F-6-P) into fructose 1,6-bisphosphate (F-1,6-BP) [106]. However, fructose-2,6-bisphosphate (F-2,6-BP) acts as the most potent allosteric activator of PFK-1 [107]. Therefore, the rate of PFK-1 is especially influenced by F-2,6-BP, whose level is closely related to 6-phosphofructo-2-kinase/fructose-2,6-bisphosphatase 3 (PFKFB3), an enzyme that is responsible for the production and consumption of F-2,6-BP [108]. Hence, the modulation of PFKFB3 has been identified as a strategy to inhibit glycolysis. And, 3-(3-Pyridyl)−1-(4-pyridyl)−2-propen-1-one (3PO) inhibits PFKFB3, thereby suppressing glycolysis and tumor growth. However, its application is limited by poor water solubility and high preclinical effective dosage. Hence, Younsoo Bae et al. prepared poly(ethylene glycol)-poly(aspartic acid) (PEG-p(ASP)) and its hydrazide derivative (PEG-p(HYD)) block copolymers. The nanomedicines were synthesized by covalently conjugating 3PO to PEG-p(HYD) via acid-sensitive hydrazone bonds. With a small particle size (38 nm), the nanomedicine accumulates in tumor cells to specifically inhibit tumor glycolysis [109].

#### Nanomedicine for the modulation of pyruvate kinase M2

The third glycolytic rate-limiting step involves pyruvate kinase (PK)-mediated irreversible conversion of phosphoenolpyruvate to pyruvate with concomitant ATP generation from ADP [110]. This irreversible reaction is critical for tumor cell bioenergetics. Among PK isoforms, PKM2 is particularly significant in malignancies, where its overexpression supports biosynthetic precursor generation in proliferating cells. Consequently, PKM2 represents a promising therapeutic target for suppressing tumor glycolytic flux.

As a naphthoquinone active compound extracted from Lithospermum species (e.g., L. erythrorhizon, Onosma paniculatum), shikonin is traditionally used for its anti-inflammatory, antimicrobial, and wound-healing properties. Recent studies demonstrate that it binds to the allosteric site of PKM2, disrupting its tetramer-dimer equilibrium. This promotes enzyme dissociation into low-activity dimers/monomers, effectively inhibiting PKM2 activity and thereby modulating glycolytic flux. Given the overexpression of PKM2 in colorectal cancer (CRC) and colorectal cancer liver metastasis (CRLM), Huang et al. selected shikonin as a PKM2 inhibitor. To address its intratumoral delivery limitations, they constructed nanomedicines (SHK@HA-MPDA) by loading shikonin onto mesoporous polydopamine nanoparticles, followed by surface modification with tumor-targeting hyaluronic acid [111]. SHK@HA-MPDA effectively inhibited cytoplasmic PKM2 tetramerization and attenuated its nuclear translocation, significantly reducing lactate and ATP production in CT26 cells. This study established an efficient, low-toxicity targeted therapeutic strategy for CRLM with synergistic potential for combination with immune checkpoint inhibitors.

Moreover, Qian et al. used photothermal agent Prussian blue nanoparticles (MPB NPs) as the nanocore, loaded with 3BP, and then encapsulated with engineered cell membranes. The cell membrane was genetically edited to highly express macrophage signal regulatory protein α (MSIRPα), which enhanced binding to CD47 on CRC cell surfaces, blocked the “don’t eat me” signal, and promoted macrophage phagocytosis. As a result, MPB-3BP@CM NPs significantly reduced intracellular ATP and lactate levels in HCT116 cells, disrupted the tricarboxylic acid cycle, and induced metabolic disorder and starvation-induced apoptosis [112].

PKM2 exhibits distinct oligomeric states with differential biological activities, of which its dimeric form displays low enzymatic activity, promotes metabolic dysregulation, and undergoes nuclear translocation for facilitating transcriptional co-activation, whereas the tetrameric state with high enzymatic activity suppresses the Warburg effect. Consequently, strategies promoting the transition of PKM2 from dimers to tetramers can effectively inhibit glycolysis, thereby starving tumor cells. For example, Xu et al. developed a PKM2 allosteric converter (PAC) comprising three functional modules: (1) an allosteric effector module featuring a serine-based motif that bound to PKM2 at Leu-353 and Asp-354, inducing dimer-to-tetramer conversion; (2) a self-assembly module containing the KLVFF peptide (derived from amyloid-β) that drives nanofiber formation; (3) an aggregation-induced emission signal module enabling real-time fluorescence imaging to track in vivo distribution and retention of PAC [113]. This study pioneered an in vivo self-assembly strategy to develop a PKM2 allosteric converter, dually suppressing the Warburg effect and chemoresistance in renal cell carcinoma (RCC) through an “enzyme-responsive self-assembly” and “sustained activation” mechanism. The PAC nanosystem integrated tumor targeting, sustained activation capability, and biocompatibility, establishing a new paradigm for nanotherapeutic intervention in RCC.

Silencing PKM2 via siRNA inhibits tumor glycolytic metabolism, thereby disrupting energy supply to cancer cells. For example, Yin et al. designed multivalent helical polypeptides (DPP) as siRNA carriers, combined with the photothermal reagent indocyanine green (ICG) and human serum albumin (HSA) to construct nanocomposites (D-I/P@HSA NCs). In MCF-7 cells, D-I/P@HSA NCs achieved 75% PKM2 silencing efficiency at the mRNA level, significantly inhibiting glycolytic metabolism with a 60% decrease in ATP [114]. In MCF-7 tumor-bearing nude mice, D-I/P@HSA NCs significantly suppressed tumor growth and enabled a 100% survival rate within a 50-day observation period. Moreover, chemoresistance in cancer leads to treatment failure, with the overexpression of ATP-binding cassette (ABC) transporters being one of the primary causes. Since ABC transporters require energy from the glycolytic process for drug efflux, siRNA-mediated silencing of PKM2 to block glycolysis can effectively reverse chemoresistance. Hence, Chen et al. developed a nanocomposite (DD-H/P NCs) based on spherical helical polypeptides (DPP), which simultaneously loaded PKM2 siRNA (siPKM2) and doxorubicin (DOX), and was further modified with hyaluronic acid (HA) for tumor targeting. DD-H/P NCs could provide PKM2 silencing efficiency of 75%, significantly reduce intracellular ATP levels by 60%, inhibit ABC transporter-mediated DOX efflux, and enhance DOX accumulation in drug-resistant tumor cells. In A549/ADR (DOX-resistant human lung adenocarcinoma cell) tumor-bearing nude mice, administration of DD-H/P NCs completely inhibited tumor growth, prolonged survival with an 80% survival rate, and significantly increased DOX accumulation in tumor sites compared with the free drug group, with no obvious toxicity in major organs [115]. Table 1 compared different nanoplatforms, their therapeutic targets (such as GLUT inhibition, glycolysis enzyme modulation, and GOx delivery), and their in vivo outcomes, to enhance clarity and provide a more structured overview.

#### Limitations and challenges

While outlining nanomedicine strategies along the glucose metabolic cascade, it is necessary to critically evaluate their relative strengths, limitations, and practical constraints. Approaches that inhibit glucose transporters (such as GLUT1 or GLUT3) offer upstream control of nutrient supply but often face compensatory upregulation of alternative transporters and may trigger systemic toxicity due to the essential role of glucose uptake in normal tissues. Enzyme-targeted interventions, including HK2, PFK1, or PKM2 modulation, enable more precise pathway interference; however, their efficacy is frequently influenced by tumor-specific metabolic heterogeneity and the ability of cancer cells to shift toward glutaminolysis or fatty-acid oxidation.

GOx-based starvation therapies provide strong and rapid metabolic pressure but are hindered by hypoxia, limited oxygen availability, and potential accumulation of H₂O₂. In contrast, multi-pathway blockade offers improved robustness against metabolic plasticity but inevitably increases formulation complexity and raises concerns regarding biosafety and pharmacokinetics. Although GOx delivery has demonstrated promising antitumor potential, the therapeutic efficacy of GOx monotherapy remains limited [116–118].

#### Combining glucose metabolic regulation with other therapies

Aberrant glucose metabolism is a hallmark of tumors, contributing to immune evasion, rapid proliferation, and therapeutic resistance. Glycolysis-driven metabolic reprogramming not only ensures a continuous supply of energy for tumor cells but also profoundly affects the TME by modulating immune responses, oxygen availability, and cell death pathways [121]. Therefore, targeting glucose metabolism holds promise not only for directly suppressing tumor growth but also for enhancing the efficacy of other therapeutic modalities through metabolic synergy or mechanistic complementation [122]. In recent years, combining glucose metabolism regulation with conventional therapies, immunotherapy, physical ablation strategies, and microenvironment modulation has emerged as a promising approach in cancer treatment. This section systematically summarizes and categorizes these combination strategies based on therapeutic type.

#### Combining glucose metabolic regulation with traditional therapies

Chemotherapy and radiotherapy remain the cornerstone treatments f or various solid tumors in clinical practice. However, tumor adaptive regulation and acquired resistance to conventional hemotherapy and radiotherapy consistently limit their efficacy and are often accompanied by severe side effects [123, 124]. Emerging evidence indicates that aberrant glucose metabolism contributes to therapeutic resistance by safeguarding pyrimidine metabolism, promoting intracellular lactate accumulation [125]. Therefore, modulating glucose metabolism can enhance the sensitivity and efficacy of traditional treatments by weakening tumor cell tolerance at an energetic level and improving hypoxic microenvironment.

Hence, recent studies have extensively explored the combination of glucose metabolism modulation with chemotherapy. In particular, glycolysis inhibition has been shown to reduce lactate accumulation and alleviate the immunosuppressive TME, thereby enhancing the cytotoxic effects of chemotherapeutic agents [126]. For this purpose, a self-delivering nanoplatform was engineered via the self-assembly of a bromodomain protein 4 (BRD4)-targeting proteolysis-targeting chimera (PROTAC) and doxorubicin (DOX) using DSPE-PEG2000 [127]. This nano-PROTACs effectively suppressed aerobic glycolysis by downregulating cellular myelocytomatosis viral oncogene homolog (c-Myc, a critical regulator of glucose metabolism), and concurrently inhibited the expression of programmed death-ligand 1 (PD-L1), thereby improving the chemotherapeutic effect of DOX. The combination therapy resulted in an 84.6% reduction in tumor weight, significantly outperforming monotherapies. Furthermore, its potent tumor suppressive activity was further validated in a mouse model of colorectal cancer with pulmonary metastasis.

In addition to its combination with chemotherapy, glucose metabolism modulation is also being explored as a strategy to augment radiotherapy. Radiotherapy efficacy is critically dependent on tumor oxygenation. However, enhanced glycolysis in tumors leads to lactate accumulation and an acidic microenvironment, which exacerbates hypoxia and ultimately impairs treatment outcomes. Hence, blocking glycolysis to reverse the hypoxic TME could help to enhance the therapeutic efficacy of chemotherapy. For example, in a study by Dong et.al, a fluorinated calcium carbonate nanocomposite (PFCE@fCaCO3-PEG) was fabricated by coating amorphous CaCO3 with dopamine-grafted perfluorosebacic acid (DA2-PFSEA) as an organic ligand via a surface-protected etching method in the presence of iron ions, followed by PEGylation, aiming to reverse tumor hypoxia and acidity-induced resistance to radiotherapy [128]. In contrast to the limited therapeutic gains observed with fCaCO3-PEG + X-ray irradiation (glucose metabolic regulation alone) or PFC@fSiO2-PEG + X-ray exposure (hypoxia attenuation alone) compared to conventional radiotherapy, PFCE@fCaCO3-PEG markedly enhanced radiotherapy efficacy, resulting in a cure for 4 of 5 mice.

Although the combination of glucose metabolism modulation with chemotherapy or radiotherapy holds significant therapeutic potential, it is hindered by critical challenges. Firstly, the therapeutic efficacy is usually limited by the metabolic plasticity and resistance of tumor cells. When blocking glucose metabolism, tumor cells rapidly adapt to metabolic inhibition by switching energy pathways (e.g., glutaminolysis or fatty acid oxidation), limiting the efficacy of glycolysis inhibitors like 2-DG or HK2-targeting agents [129]. In addition, tumor cells can also very intelligently activate energy compensation upon glycolysis inhibition. Hypoxic regions in tumors exhibit radioresistance and may upregulate alternative metabolic enzymes, reducing the radiosensitizing effects of glycolysis blockade [130]. Then, the combination of glucose metabolism modulation with chemotherapy or radiotherapy faces the limitation of nonspecific toxicity and off-target effects. For example, systemic inhibition of glycolysis can impair normal tissues with high glucose dependence, such as brain and heart, exacerbating chemotherapy-induced toxicity, especially cardiotoxicity from anthracyclines. Radiotherapy-induced inflammation may further disrupt glucose homeostasis, increasing the risk of metabolic complications, such as hyperglycemia and lactic acidosis [131]. Moreover, the difficulty also lies in achieving precise control over drug release kinetics and dosage.

#### Limitations and challenges

Although combining glucose metabolism regulation with traditional therapies such as chemotherapy and radiotherapy can enhance antitumor efficacy, this strategy also introduces several limitations. Metabolic inhibition may exacerbate the systemic toxicity of cytotoxic drugs by weakening the stress tolerance of normal proliferative tissues. In addition, the timing, dosing sequence, and pharmacokinetic matching between metabolic modulators and conventional therapeutics remain difficult to optimize. Importantly, metabolic suppression may impair the function of normal immune and hematopoietic cells, potentially increasing treatment-related adverse effects. These challenges underscore the need for carefully designed scheduling strategies and tumor-selective delivery systems to balance efficacy and safety.

#### Combining glucose metabolic regulation with immunotherapy

Significant advances in cancer immunotherapy have been realized with the clinical implementation of immune checkpoint inhibitors (ICIs). Nevertheless, the immunosuppressive TME remains a major barrier limiting the efficacy and response rates of such therapies. Emerging evidence suggests that glucose metabolic regulation plays a pivotal role in modulating the TME by alleviating extracellular acidification and improving immune cell function, thereby overcoming immune tolerance and enhancing the therapeutic outcomes of immunotherapy [132].

**Fig. 7:**
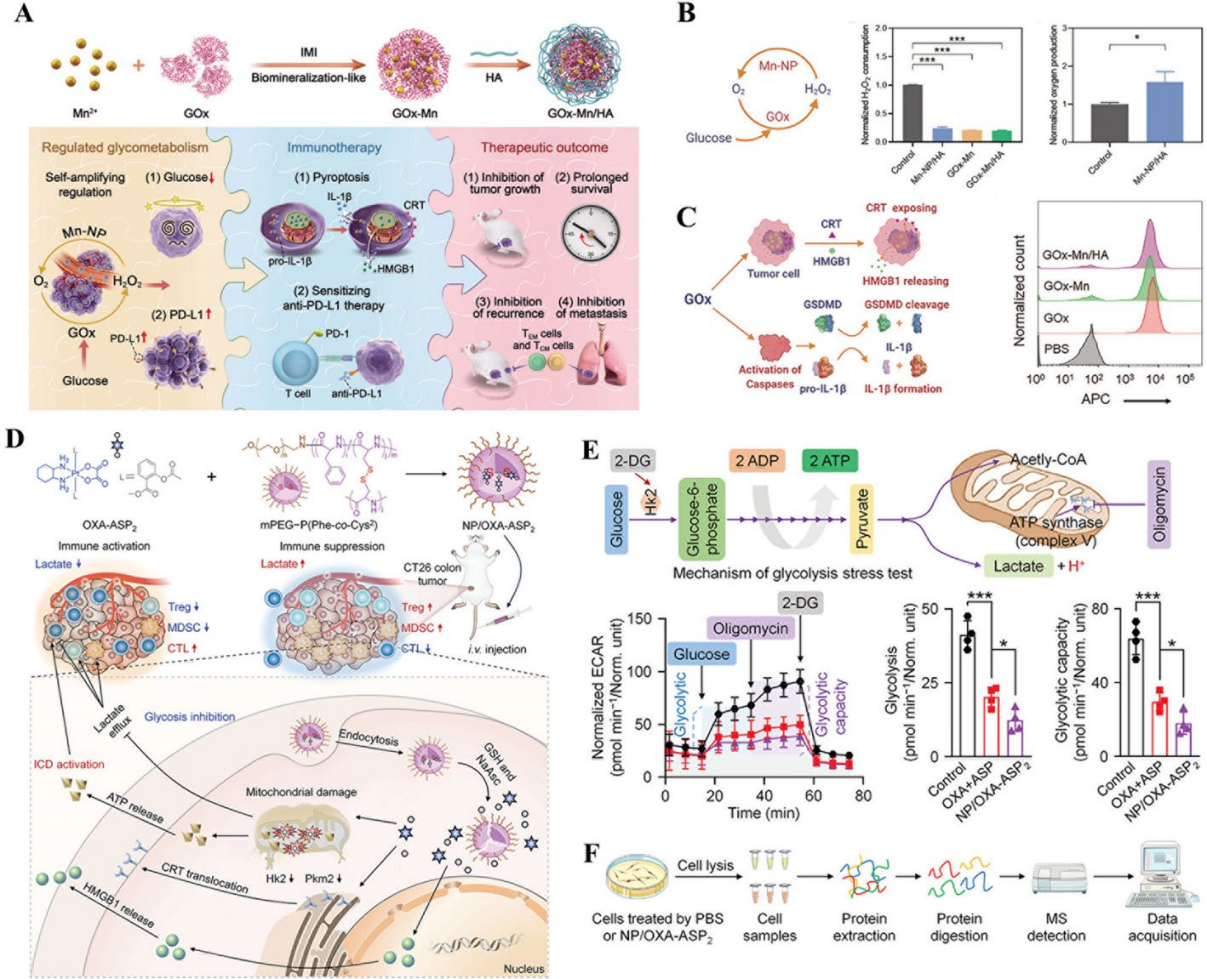
Nanomedicine for combining glucose metabolic regulation with immunotherapy. (**A**) Schematic diagram of GOx-Mn/HA design and action mechanism. (**B**) GOx-Mn/HA coordinated two synergistic enzymatic functions. (**C**) GOx-Mn/HA induced immunogenic signaling molecule release by rewiring glycometabolic pathways and triggering pyroptotic cell death. [133] Copyright © 2022 Wiley. (**D**) Schematic diagram of NP/OXA-ASP design, in vivo transport, and action mechanism. (**E**) Glycolytic stress assays demonstrated that NP/OXA-ASP impaired glucose-to-lactate conversion efficiency by inhibiting key glycolytic enzymes and mitochondrial respiration. (**F**) Proteomic profiling revealed the synergistic antitumor mechanism of NP/OXA-ASP. *p < 0.05, and ***p < 0.001. [137] Copyright © 2025 Wiley

Inspired by this, GOx delivery has been explored for tumor starvation therapy through glucose depletion. A biomineralization strategy was employed to construct dual-enzyme hybrid nanoparticles (GOx-Mn/HA) for glucose depletion, with hyaluronic acid coating enabling targeted delivery to breast cancer cells [133]. As shown in Fig. 7A, biomineralized GOx-Mn/HA were synthesized through coordination-driven self-assembly between GOx and manganese ions, followed by surface functionalized with HA via electrostatic adsorption. Within tumors, GOx-Mn/HA released GOx to deplete glucose while simultaneously liberating manganese-based nanoparticles (Mn-NPs). The Mn-NPs catalyzed H₂O₂ decomposition into O₂, enhancing GOx catalytic efficiency and establishing a self-amplifying feedback loop. Concerted glucose deprivation and oxidative stress induce tumor cell pyroptosis, releasing damage-associated molecular patterns and upregulating programmed death-ligand 1 (PD-L1) expression on tumor cells, boosting a robust antitumor immune response. GOx-Mn/HA coordinated two synergistic enzymatic functions: i) GOx initiated substrate depletion by converting glucose to gluconic acid with concomitant H₂O₂ production; ii) Mn-NPs subsequently utilized the generated H₂O₂ as feedstock for catalytic oxygen evolution, establishing a self-sustaining biochemical cycle (Fig. 7B). Moreover, GOx-Mn/HA primarily induced immunogenic signaling molecule release by rewiring glycometabolic pathways and triggering pyroptotic cell death (Fig. 7C). When combined with anti-PD-L1 therapy, GOx-Mn/HA provided an enhanced tumor inhibition rate of 92.9%, surpassing that of GOx-Mn/HA alone.

Inspired by this, GOx delivery has been explored for tumor starvation therapy through glucose depletion. A biomineralization strategy was employed to construct dual-enzyme hybrid nanoparticles (GOx-Mn/HA) for glucose depletion, with hyaluronic acid coating enabling targeted delivery to breast cancer cells [133]. As shown in Fig. 7A, biomineralized GOx-Mn/HA were synthesized through coordination-driven self-assembly between GOx and manganese ions, followed by surface functionalized with HA via electrostatic adsorption. Within tumors, GOx-Mn/HA released GOx to deplete glucose while simultaneously liberating manganese-based nanoparticles (Mn-NPs). The Mn-NPs catalyzed H₂O₂ decomposition into O₂, enhancing GOx catalytic efficiency and establishing a self-amplifying feedback loop. Concerted glucose deprivation and oxidative stress induce tumor cell pyroptosis, releasing damage-associated molecular patterns and upregulating programmed death-ligand 1 (PD-L1) expression on tumor cells, boosting a robust antitumor immune response. GOx-Mn/HA coordinated two synergistic enzymatic functions: i) GOx initiated substrate depletion by converting glucose to gluconic acid with concomitant H₂O₂ production; ii) Mn-NPs subsequently utilized the generated H₂O₂ as feedstock for catalytic oxygen evolution, establishing a self-sustaining biochemical cycle (Fig. 7B). Moreover, GOx-Mn/HA primarily induced immunogenic signaling molecule release by rewiring glycometabolic pathways and triggering pyroptotic cell death (Fig. 7C). When combined with anti-PD-L1 therapy, GOx-Mn/HA provided an enhanced tumor inhibition rate of 92.9%, surpassing that of GOx-Mn/HA alone.

In addition to enhancing glucose consumption through GOx, glucose uptake can also be attenuated by downregulating GLUT1. Based on this strategy, a Mn-based galvanic cell (MnG) was designed to downregulate GLUT1 expression and reprogram tumor glucose metabolism [135]. This modulation subsequently led to the downregulation of three prime repair exonuclease 2, thereby activating the cGAS-STING pathway and mitigating immunotherapy resistance. In vivo studies demonstrated that the MnG combined with anti-PD-L1 (αPD-L1) therapy significantly enhanced dendritic cell maturation in lymph nodes and cytotoxic T lymphocyte infiltration, compared to αPD-L1 treatment alone. Moreover, in a rabbit orthotopic liver cancer model, MnG-lipiodol dispersion resulted in more extensive tumor damage and reduced proliferative activity relative to clinically approved lipiodol. Following the same strategy, a multifunctional metabolic nanoregulator (D/B/CQ@ZIF-8@CS) was developed by co-encapsulating 2-deoxy-D-glucose, GLUT1 inhibitor, and chloroquine into chondroitin sulfate-modified zeolitic imidazolate framework-8 [136]. This system simultaneously inhibited glycolysis, glucose uptake, and autophagic flux, thereby comprehensively disrupting the tumor's energy supply and survival mechanisms. When combined with anti-cytotoxic T-lymphocyte-associated protein 4 (anti-CTLA-4) therapy, it significantly increased the proportion of cytotoxic T cells, while reducing Foxp3 Treg cells to half the level observed with D/B/CQ@ZIF-8@CS monotherapy. These results indicated that the combination therapy effectively relieved immune suppression and enhanced antitumor immune responses.

Aberrant glucose metabolism in cancer cells fosters an immunosuppressive tumor microenvironment that suppresses T-cell function and impedes immunogenic cell death (ICD). To address these limitations, Chen et al. developed a glucose metabolism-targeted poly(amino acid) nanoplatform (NP/OXA-ASP) that enhanced the chemoimmunotherapeutic efficacy of oxaliplatin (OXA) by suppressing tumor glycolysis, triggering ICD, and reprogramming the immunosuppressive niche [137]. For preparation, OXA was first conjugated to aspirin (ASP) via ester linkages to form the OXA-ASP prodrug. Subsequently, an amphiphilic poly(amino acid) copolymer, mPEG-P (Phe-co-Cys₂), was synthesized. Finally, the OXA-ASP prodrug was encapsulated within the polymeric carrier using nanoprecipitation (Fig. 7D). In tumor, the released OXA induced immunogenic cell death (ICD), while ASP suppressed glycolysis. This dual action effectively reprogrammed the immunosuppressive TME, achieving potent synergy between tumor metabolic modulation and chemoimmunotherapy. Glycolytic stress assays demonstrated that NP/OXA-ASP impaired glucose-to-lactate conversion efficiency by inhibiting key glycolytic enzymes and mitochondrial respiration, thereby reducing extracellular acidification rate values (Fig. 7E). Moreover, proteomic profiling revealed that the NP/OXA-ASP synergistically combated tumors through a triple-barreled mechanism of metabolic suppression, apoptosis induction, and immune activation, rather than operating via single-target effects (Fig. 7F). In a CT26 tumor-bearing mouse model, the nanomedicine prolonged the drug circulation half-life (β phase t₁/₂ = 29.2 hours) and achieved a tumor inhibition rate of 85.2%, outperforming the combination of free drugs.

Although the combination of glucose metabolism and immunotherapy has more advantageous and clinical translation potential, it still faces the following challenges. Firstly, the differences in metabolic preferences among different tumor subclones, such as glycolytic dependent type and oxidative phosphorylation-dominated type, lead to the failure of a single metabolic intervention strategy [138]. For example, in PD-1 resistant patients, the glycolytic active subgroup escapes immune killing by up-regulating LDHA [139]. Then, the dynamically changing TME during the treatment process weakens the therapeutic effect. For instance, T cell infiltration caused by immunotherapy can reshape the metabolic microenvironment (such as IFN-γ driving IDO expression), offsetting the effect of metabolic intervention [140]. Moreover, the combination of glucose metabolism drugs and immunoregulatory drugs faces difficulties in co-delivery, including spatiotemporal synergy problems. For example, the co-loaded nanoparticles of anti-PD-L1 antibodies and 2-DG have a lower therapeutic effect than sequential administration due to the mismatch of release kinetics. The difficulties in targeting drug delivery will further counteract the therapeutic effect. For example, metabolic regulation requires simultaneous targeting of tumor cells and immune cells, but existing targeting ligands may cause side effects. 

Therefore, the development of next-generation drug delivery systems must prioritize spatiotemporal coordination to maximize metabolic-immunotherapy synergy. A promising strategy involves engineering sequentially responsive nanoplatforms capable of recruiting immune cells before triggering the controlled release of metabolic modulators, thereby aligning drug action with immunological processes. Furthermore, advancing cell-selective targeting is critical to mitigate off-target toxicity through dual-ligand systems or microenvironment-specific activation. These innovations must be coupled with real-time monitoring technologies (e.g., PET tracers for glucose uptake) to dynamically assess target engagement and therapeutic windows.

#### Limitations and challenges

Although the combination of glucose metabolic regulation and immunotherapy shows synergistic potential, several limitations remain. Because glycolysis is also essential for the activation and effector function of T cells and NK cells, nonspecific metabolic inhibition may impair antitumor immunity. Moreover, metabolic heterogeneity within the tumor microenvironment results in complex nutrient competition between tumor and immune cells, complicating selective targeting. The immunogenicity and long-term biosafety of enzyme-based or metabolic nanomedicines also require further investigation.

#### Combining glucose metabolic regulation with programmed cell death

**Fig. 8:**
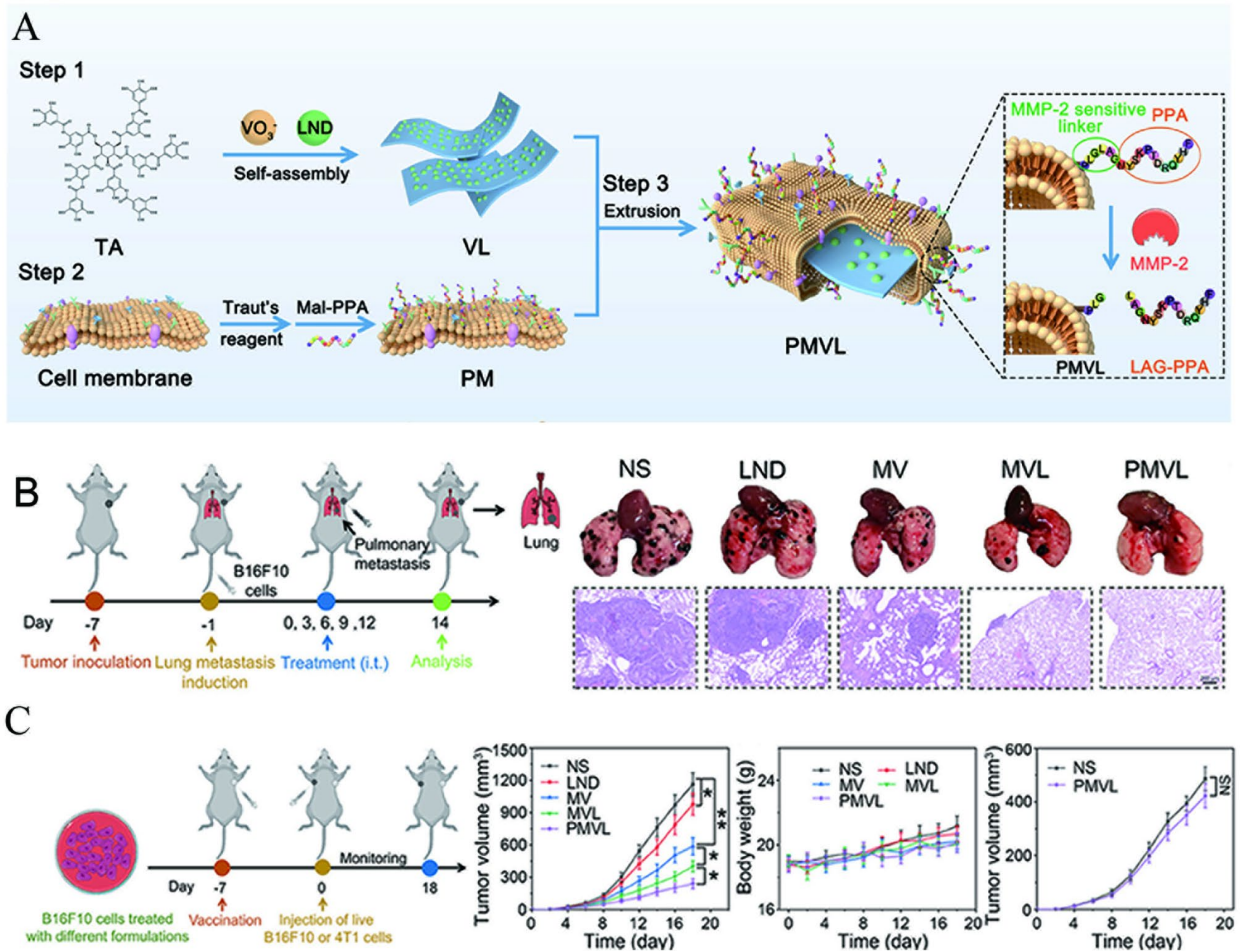
Nanomedicine for combining glucose metabolic regulation with programmed cell death. (**A**) Schematic diagram of PMVL design and preparation process. (**B**) Schematic diagram of B16F10 pulmonary metastasis mice model and the results showed that PMVL could inhibit lung metastases. (**C**) Results showed that PMVL-induced tumor vaccine could inhibit heterologous 4T1 tumor growth in immunized mice. *p < 0.05, and **p < 0.01. [144] Copyright © 2023 American Chemical Society

Programmed cell death (PCD) is a tightly regulated process in multicellular organisms that eliminates superfluous or abnormal cells through various forms, including apoptosis, ferroptosis, cuproptosis, and pyroptosis. While apoptosis has traditionally been the focus of PCD, emerging forms such as ferroptosis, cuproptosis, and pyroptosis have garnered increasing attention in the context of cancer [141, 142]. Notably, targeting glycolysis represents a promising approach to induce these alternative PCD pathways by promoting the accumulation of ROS and suppressing the generation of Nnicotinamide adenine dinucleotide phosphate (NADPH), thereby providing a rationale for combination therapies in cancer treatment [143]. Based on this, a nanoplatform (PMVL) was developed by loading lonidamine (LND) onto tannic acid-coordinated vanadium oxides and camouflaging them with PD-L1 inhibitory peptides modified tumor cell membranes [144]. As shown in Fig. 8A, VL nanosheets were self-assembled via coordination between tannic acid and LND under ammonium metavanadate induction. Subsequently, B16F10 tumor cell membranes were extracted, thiolated, and conjugated with PPA peptide. Finally, PMVL nanoparticles that possessed matrix metalloproteinase (MMP) responsiveness were fabricated by extruding the modified membranes to coat VL nanosheets. The valence-cycling behavior of vanadium facilitated ferroptosis, while LND inhibited glycolysis and the pentose phosphate pathway by reducing ATP and NADPH levels, thereby amplifying ferroptotic cell death. In a B16F10 pulmonary metastasis model, PMVL treatment not only potently suppressed primary tumor growth but also significantly inhibited lung metastases (Fig. 8B). Innovatively, the authors employed PMVL-pretreated tumor cells as a vaccine, which effectively inhibited heterologous 4T1 tumor growth in immunized mice (Fig. 8C).

To enhance ferroptosis-based cancer therapy via glucose metabolism modulation, dopamine (DA) and Fe ions were co-coordinated to encapsulate bacterial outer membrane vesicles (OMVs), followed by in situ growth of ultrasmall gold nanoparticles (Au NPs) on the surface to construct a multifunctional nanoplatform (OMV-DFA) [145]. The Au NPs catalyzed glucose consumption and elevated H₂O₂ levels, which then reacted with released Fe ions to induce ferroptosis and immunogenic cell death. Although OMV-DaFe (ferroptosis alone) achieved a tumor inhibition rate of approximately 60%, all treated mice died within 40 days. In contrast, OMV-DFA treatment nearly halted tumor growth and achieved a 50-day survival rate of 83.3% in tumor-bearing mice. These studies underscored the therapeutic potential of combining glucose metabolism disruption with ferroptosis to achieve enhanced antitumor efficacy and prolonged survival.

To enhance ferroptosis-based cancer therapy via glucose metabolism modulation, dopamine (DA) and Fe ions were co-coordinated to encapsulate bacterial outer membrane vesicles (OMVs), followed by in situ growth of ultrasmall gold nanoparticles (Au NPs) on the surface to construct a multifunctional nanoplatform (OMV-DFA) [145]. The Au NPs catalyzed glucose consumption and elevated H₂O₂ levels, which then reacted with released Fe ions to induce ferroptosis and immunogenic cell death. Although OMV-DaFe (ferroptosis alone) achieved a tumor inhibition rate of approximately 60%, all treated mice died within 40 days. In contrast, OMV-DFA treatment nearly halted tumor growth and achieved a 50-day survival rate of 83.3% in tumor-bearing mice. These studies underscored the therapeutic potential of combining glucose metabolism disruption with ferroptosis to achieve enhanced antitumor efficacy and prolonged survival.Disulfidptosis is a recently identified and distinct form of programmed cell death, characterized by the aberrant formation of disulfide bonds in actin cytoskeletal proteins, leading to cellular dysfunction and death [146]. In a study by Zhang et.al, FeOOH nanoshuttles (NSs) co-loaded with gold (Au) nanodots and an iron-apigenin (Ap) complex were developed to simultaneously induce disulfidptosis and ferroptosis under systemic glucose deprivation [147]. Mechanistically, Au nanodots exhibited glucose oxidase-like activity to enhance glucose consumption, while Ap suppressed glucose uptake by downregulating GLUT1 expression. This dual action induced systemic glucose deprivation, limited NADPH replenishment, and disrupted cystine/cysteine metabolism, collectively triggering disulfidptosis and ferroptosis. Compared to the FeOOH NSs group, the FeOOH@Fe-Ap@Au NSs group (combination treatment) achieved the smallest tumor volume and the highest tumor growth inhibition rate of 96.9%, demonstrating potent antitumor efficacy.

Pyroptosis is a highly inflammatory form of programmed cell death that can be triggered by a rapid surge in ROS [148]. Although tumor cells often exhibit strong resistance to oxidative stress, targeting abundant endogenous antioxidant GSH presents a viable strategy to disrupt this balance. To this end, a copper-based nano-inducer (CGBH NN) was created by incorporating GOx and buthionine sulfoximine (BSO), followed by coating with hyaluronic acid [149]. This system disrupted intracellular metabolic and redox homeostasis through GOx and Cu2+, thereby inducing pyroptosis in tumor cells. After treatment for 23 days, CT26 tumor-bearing mice receiving the combined glycolysis and pyroptosis intervention (CGBH NN) exhibited a significantly reduced average tumor volume (268 mm³) and a markedly extended survival time compared to all other groups, highlighting the therapeutic potential of simultaneously targeting metabolic and oxidative stress for enhanced pyroptosis efficacy.

The integration of glucose metabolism-targeting approaches with PCD pathways holds promise for enhancing tumor elimination. However, several key challenges hinder its clinical translation. Firstly, most of the tumor cells present metabolic plasticity and resistance to PCD induction. Tumor cells under glucose deprivation may upregulate alternative energy pathways, such as glutaminolysis or fatty acid oxidation, to evade apoptosis or ferroptosis. For example, inhibition of glycolysis via 2-DG can activate AMP-activated protein kinase, promoting autophagy as a survival mechanism [150]. Secondly, conflicting effects are presented in PCD pathways. For example, glycolysis inhibitors can impair ATP production, preventing caspase-dependent apoptosis as metabolic dormancy [151]. Glucose metabolism supports glutathione peroxidase (GPX4) synthesis (a ferroptosis suppressor). Overly aggressive glycolysis blockade may upregulate GPX4, counteracting ferroptosis inducers [152]. More importantly, the delivery barrier is the major problem for the combination of glucose metabolism and PCD. PCD inducers may require glucose starvation before or during treatment, but optimal sequencing remains unclear. Hence, co-delivering metabolic drugs and PCD activators demands stimuli-responsive systems.

#### Limitations and challenges

Despite the rationale for integrating glucose metabolic regulation with programmed cell death, several limitations should be considered. Energy depletion can differentially affect tumor and normal cells, raising concerns about off-target cytotoxicity. Moreover, excessive metabolic stress may trigger adaptive survival pathways, such as autophagy or stress-response signaling, which can attenuate the intended cell-death response. The efficiency of inducing ferroptosis, cuproptosis, or pyroptosis also depends on tumor redox status and metal ion availability, which vary substantially across tumor types and stages. Furthermore, the spatiotemporal control of death induction remains challenging, emphasizing the need for tumor-selective activation and finely tuned release kinetics. 

The long-term biosafety of metal-based nanomedicines remains a concern due to the potential accumulation of metals in organs like the liver, spleen, and brain, which may lead to dose-dependent neurotoxicity and hepatotoxicity. To address these issues, strategies such as surface coatings, chelators, and biodegradable architectures have been proposed. Surface coatings can reduce direct interactions with biological systems, while chelators can bind metal ions, preventing toxicity. Additionally, biodegradable nanoplatforms can degrade over time, reducing metal accumulation. These mitigation strategies help improve the safety, but they may not completely resolve the long-term safety concerns.

#### Combining glucose metabolic regulation with energy metabolism regulation

Tumor cells are characterized by metabolic abnormalities, with mitochondria serving as both the energy powerhouse and central hub of cellular metabolism. As such, mitochondria have emerged as attractive targets for regulating cellular bioenergetics in cancer therapy [153]. Glycolysis and mitochondrial OXPHOS are the two primary pathways through which tumors generate energy. Due to metabolic heterogeneity and compensatory mechanisms, tumor cells often shift their metabolic phenotype from OXPHOS to glycolysis to evade mitochondrial-targeted therapies. Therefore, the simultaneous targeting of OXPHOS and glycolysis represents a promising strategy for antitumor bioenergetic therapy through synergistic mechanisms [154]. Inhibition of oxidative phosphorylation is primarily achieved by disrupting the mitochondrial membrane potential or blocking the electron transport chain. Based on this, a glycopolymer (GP) containing a caged hydrogen sulfide (H2S)/H2O2 dual-donor was synthesized to wrap GOx, aiming for complete depletion of tumor energy sources [155]. Following intravenous injection, GP acted as a GLUT1-targeting ligand to enhance the delivery of GOx. The released thio-glucose was catalyzed by GOx into cytotoxic H₂S while preserving H2O2, leading to disruption of the mitochondrial electron transport chain and dissipation of the mitochondrial membrane potential. Concurrently, GOx effectively depleted endogenous glucose, thereby inhibiting glycolysis. In 4T1-tumor-bearing mice model, tumors treated with single mitochondrial metabolic modulation (BP) exhibited growth trends similar to the PBS control group, with an average volume exceeding 900 mm³. In contrast, the GP-treated group showed the smallest tumor volumes, with a tumor growth inhibition rate of 80.0%, indicating significant suppression of tumor progression. Therefore, simultaneous inhibition of both OXPHOS and glycolysis represents a promising strategy for antitumor bioenergetic therapy via synergistic mechanisms.

Building upon these efforts to remodel tumor energy metabolism, further strategies have focused on reprogramming glucose utilization from glycolysis to OXPHOS by targeting key metabolic regulators such as pyruvate dehydrogenase (PDH). PDH is a key metabolic switch that determines whether pyruvate enters the TCA cycle for mitochondrial oxidation or is converted to lactate. Dichloroacetate (DCA), a metabolic modulator with anticancer properties, promotes PDH activation; however, its efficacy is often compromised by the hypoxic TME. To address this limitation, ultrasmall (<6 nm) DCA-conjugated manganese ferrite nanoparticles (MnFe₂O₄-DCA) were developed to modulate tumor glucose metabolism and ATP catabolism [156]. Upon intravenous administration, their ultrasmall size facilitated efficient mitochondrial entry and robust PDH activation. MnFe₂O₄ also decomposed endogenous H₂O₂, generating oxygen to enhance the bioactivity of DCA under hypoxic conditions. Compared to either DCA or MnFe₂O₄ alone, MnFe₂O₄-DCA significantly downregulated CD39 and CD73 expression, thereby inhibiting extracellular ATP degradation and reversing the tumor immune microenvironment (TIME). Notably, MnFe₂O₄ alone had minimal impact on pyruvate dehydrogenase kinase 1 (PDK1) and phosphorylated PDH (p-PDH), while MnFe₂O₄-DCA significantly reduced their expression, matching the effect of a 100-fold higher dose of free DCA.

In addition to glucose, glutamine metabolism also supports the energy production and biosynthetic demands of highly proliferative cancer cells, with both pathways capable of compensatory switching depending on cellular conditions. Although numerous glycolysis-targeting strategies have been widely developed for tumor starvation therapy, they often neglect the compensatory role of glutamine metabolism. Even under glycolytic inhibition, glutamine can be converted into glutamate by glutaminase (GLS1) and subsequently into α-ketoglutarate via two enzymatic steps, replenishing carbon intermediates in the TCA cycle [157, 158]. This metabolic flexibility underscores the need for co-targeting both glucose and glutamine pathways. GLS1, a key enzyme in glutamine metabolism, is frequently overexpressed in various tumor types, making it a common target for glutamine deprivation strategies [159]. However, the effectiveness of GLS1 inhibitors is often limited by poor tumor penetration and low bioavailability. To tackle these obstacles, a functional metalorganic framework (MOF)-based nanoreactor (PCP-MOF@G@B) was designed, comprising a pH-responsive detachable copolymer shell and a ROS-sensitive degradable MOF core for co-delivery of GOx and the GLS1 inhibitor BPTES [160]. Following intravenous injection, the nanoplatform achieved stepwise drug delivery through effective blood circulation, tumor penetration, and cellular uptake, facilitated by the protective polymer shell, acidic TME activation, and ROS-triggered drug release. Therapeutic evaluation demonstrated that PCP-MOF@G@B significantly inhibited tumor growth, resulting in the smallest tumor volumes and prolonged survival of tumor-bearing mice up to 45 days, which markedly outperformed the PCP-MOF@G group that targeted glucose metabolism alone.

Despite the promising potential of combining glucose metabolism modulation with mitochondrial-targeted therapies for cancer treatment, several challenges remain. A major obstacle is the heterogeneity of tumors and the metabolic plasticity of cancer cells. Some tumors may develop resistance by activating alternative metabolic pathways, such as fatty acid oxidation, serine metabolism, or the pentose phosphate pathway, to sustain energy production [161]. Moreover, since normal cells also rely on OXPHOS for energy, off-target effects may lead to irreversible damage and unintended alterations in immune cell function. Therefore, elucidating the mechanisms underlying therapeutic resistance and affecting immunometabolism remains a critical priority. Spatiotemporally specific interventions targeting metabolic pathways, along with strategies to enhance drug specificity toward tumor cells, are essential for improving the efficacy and safety of cancer metabolism-based therapies.

#### Combining glucose metabolic regulation with physiotherapy

Physiotherapy, such as photothermal therapy (PTT) and photodynamic therapy (PDT), has garnered widespread attention for localized cancer treatment due to their spatial precision, minimal invasiveness, and controllable activation. PTT utilizes photothermal agents to convert NIR light into heat, inducing localized hyperthermia and direct tumor ablation. In addition, PDT relies on light-activated photosensitizers to generate ROS, which initiate oxidative damage and apoptosis within tumor cells [162]. These modalities not only enable site-specific tumor destruction with reduced systemic toxicity but also exhibit strong synergy with metabolic therapies, particularly those targeting glucose metabolism. For instance, ROS can impair mitochondrial function, disrupt redox balance, and further suppress glycolysis or OXPHOS pathways, thereby enhancing the efficacy of glucose deprivation strategies [163].

The integration of glucose metabolism regulation with PTT provides synergistic advantages for enhanced tumor ablation. GOx-mediated glucose depletion and H₂O₂ generation facilitate localized acidification and elevated oxidative stress, thereby amplifying therapeutic impact of PTT and reinforcing a thermo-metabolic feedback loop [164]. To overcome the limited efficacy of GOx under hypoxic conditions, a nanoplatform (OAGO) was developed by the self-assembly of GOx and oligomycin A (OA), encapsulated within OMV [25]. OA reduced intratumoral oxygen consumption, while OMV alleviated hypoxia by improving blood oxygen levels, thereby potentiating GOx activity to exacerbate energy depletion. Additionally, OAGO induced hemoglobin leakage from red blood cells, which served as a natural photothermal agent capable of absorbing NIR light and converting it into heat, thereby augmenting PTT. In a 4T1-tumor-bearing mouse model, OAGO enabled effective PTT under NIR irradiation. Without laser exposure, GOx and OA alone exhibited limited tumor inhibition rates of 24.70% and 35.83%, respectively. In contrast, OAGO achieved a suppression rate of 83.22%, and when combined with laser irradiation, is resulted in near-complete tumor ablation by day 5, demonstrating a potent antitumor effect.

Analogously, a multifunctional antitumor nanosystem (Ce-Mn)-PEI/GOx-PEG was developed based on a novel cerium-manganese heterojunction (CeO2Mn1.08OX) modified with GOx and polyethylene glycol [165]. The CeO2Mn1.08OX nanocluster exhibited catalase (CAT)-, peroxidase (POD)-, and oxidase (OXD)-like activities, which alleviated tumor hypoxia and depleted intracellular GSH in the acidic TME, thereby sensitizing tumor cells to GOx-mediated starvation therapy. In addition, its unique heterojunction structure conferred excellent photothermal properties. In a 4T1 tumor-bearing BALB/c mouse model, 14 days of treatment resulted in average tumor volumes of 67.4%, 49.5%, 25.0%, and 8.2% of the control in the (Ce-Mn)-PEI-PEG, (Ce-Mn)-PEI/GOx-PEG, (Ce-Mn)-PEI-PEG + L, and (Ce-Mn)-PEI/GOx-PEG + L groups, respectively, demonstrating a potent synergistic antitumor effect achieved by the combined therapeutic strategy.

Despite the high therapeutic efficacy of PTT, various tumor cells tend to upregulate heat shock proteins (HSPs) in response to PTT-induced thermal stress [166]. HSPs facilitate protein folding, stabilize the cytoskeleton, and inhibit apoptosis, thereby enhancing cellular thermotolerance. Additionally, PTT-induced inflammation has been shown to promote tumor cell migration, serving as a potential trigger for metastasis [167]. To address these challenges, a nanotherapeutic platform (FMyP), self-assembled from myricetin (My), iron ions, and polyvinylpyrrolidone, was developed for the combined intervention of glucose metabolism disruption, anti-inflammation, starvation, and PTT [168]. Myricetin inhibited GLUT 1 and disrupted mitochondrial function, effectively blocking tumor energy supply. The resultant energy depletion downregulated HSP expression, thereby enhancing PTT efficacy. Meanwhile, FMyP exhibited broad-spectrum ROS scavenging ability, mitigating PTT-induced inflammation. Compared to the PBS control, FMyP treatment partially suppressed tumor growth, whereas FMyP combined with laser irradiation (FMyP + L) achieved complete tumor eradication without recurrence within 14 days, demonstrating superior antitumor effects of the combinational therapy over monotherapy.

The modulation of glucose metabolism sensitizes tumor cells to PDT-induced ROS by depleting intracellular energy stores and weakening antioxidant defenses, thereby establishing a metabolism-ROS positive feedback loop [169]. However, due to the extremely short lifespan and limited diffusion radius of ROS, a mitochondria-targeted nanocomposite (DCK) was developed [170]. This nanoplatform was constructed via solid-phase peptide synthesis by sequentially linking a tumor-targeting peptide (RGD), a cell-penetrating peptide (R9), a caspase-3 (Casp3)-cleavable peptide (DEVD), a mitochondria-targeting peptide (dKLA) coupled with photosensitizer chlorin e6 (Ce6), followed by lecithin conjugation. Through electrostatic adsorption, DCK was further complexed with siRNA targeting GLUT1 to form DCK@siGLUT1. The dKLA facilitated selective mitochondrial accumulation and apoptosis induction. Subsequently, activated Casp3 cleaved the DEVD sequence, triggering the release of siGLUT1 and silencing GLUT1 expression. Meanwhile, Ce6 enhanced mitochondrial damage and metabolic disruption upon light activation. In 4T1 tumor-bearing mice, the non-mitochondrial-targeted nanocomposite negative control group (DCN) + L exhibited greater tumor inhibition than DCK@siGLUT1 alone, suggesting that PDT induced a stronger antitumor response than glucose metabolism regulation alone. Notably, the DCK@siGLUT1 + L group achieved the most effective tumor suppression, validating the synergistic advantage of combining glucose metabolism inhibition with PDT for enhanced antitumor efficacy.

The modulation of glucose metabolism sensitizes tumor cells to PDT-induced ROS by depleting intracellular energy stores and weakening antioxidant defenses, thereby establishing a metabolism-ROS positive feedback loop [169]. However, due to the extremely short lifespan and limited diffusion radius of ROS, a mitochondria-targeted nanocomposite (DCK) was developed [170]. This nanoplatform was constructed via solid-phase peptide synthesis by sequentially linking a tumor-targeting peptide (RGD), a cell-penetrating peptide (R9), a caspase-3 (Casp3)-cleavable peptide (DEVD), a mitochondria-targeting peptide (dKLA) coupled with photosensitizer chlorin e6 (Ce6), followed by lecithin conjugation. Through electrostatic adsorption, DCK was further complexed with siRNA targeting GLUT1 to form DCK@siGLUT1. The dKLA facilitated selective mitochondrial accumulation and apoptosis induction. Subsequently, activated Casp3 cleaved the DEVD sequence, triggering the release of siGLUT1 and silencing GLUT1 expression. Meanwhile, Ce6 enhanced mitochondrial damage and metabolic disruption upon light activation. In 4T1 tumor-bearing mice, the non-mitochondrial-targeted nanocomposite negative control group (DCN) + L exhibited greater tumor inhibition than DCK@siGLUT1 alone, suggesting that PDT induced a stronger antitumor response than glucose metabolism regulation alone. Notably, the DCK@siGLUT1 + L group achieved the most effective tumor suppression, validating the synergistic advantage of combining glucose metabolism inhibition with PDT for enhanced antitumor efficacy.

Despite the promising therapeutic outcomes of PTT and PDT, both modalities face safety concerns. PTT often suffers from a lack of thermal confinement and nonspecific accumulation of photothermal agents (PTAs), which may result in collateral damage to surrounding normal tissues. Additionally, tumor cells can develop thermotolerance by upregulating HSPs [172]. PDT is constrained by systemic photosensitivity, poor tissue penetration, and a strong dependence on oxygen availability, limiting its effectiveness in hypoxic tumors [173, 174]. Moreover, both therapies can induce inflammatory responses that may inadvertently promote tumor progression or metastasis. To address these challenges, advances in drug delivery systems have focused on enhancing tumor-specific accumulation and stimulus-responsive activation of photosensitizers and PTAs. Hence, reconciling targeting precision with formulation simplicity remains key to advancing clinical applicability [175].

#### Limitations and challenges

Although combined regulation of glucose and energy metabolism can induce severe bioenergetic stress, dual pathway inhibition may also impair mitochondrial function in normal tissues and increase systemic toxicity. Tumor cells may further activate alternative metabolic routes, such as fatty acid or amino acid metabolism, to escape energy collapse. In addition, precise coordination of multi-pathway inhibition in vivo remains technically challenging, underscoring the need for tumor-selective delivery and controlled release strategies.

#### Combining glucose metabolic regulation with matrix modulation

With the deepening understanding of the complexity of the TME, research focus has gradually shifted from solely targeting tumor cells to the systemic regulation of the tumor ecosystem, particularly through the intervention of matrix metabolism. Matrix cells within the TME, including cancer-associated fibroblasts (CAFs), endothelial cells, and immune cells, commonly undergo metabolic reprogramming, primarily relying on aerobic glycolysis to produce abundant lactate, ketone bodies, and non-essential amino acids. These metabolites not only supply energy and biosynthetic precursors to tumor cells but also modulate microenvironmental acidity and immunosuppression, thereby promoting tumor proliferation, invasion, and therapeutic resistance [176, 177]. Restoring the metabolic homeostasis of matrix cells by developing agents that normalize their metabolism holds promise for correcting aberrant matrix metabolism in tumors. Moreover, dual targeting of matrix metabolic pathways and tumor cell glycolytic networks can effectively disrupt tumor energy supply and metabolic symbiosis, overcoming metabolic heterogeneity and therapy resistance to enhance treatment efficacy [178, 179]. Moreover, targeting and modulating CAFs can significantly enhance the accumulation of nanomedicines within tumors, particularly in solid tumors, thereby improving the therapeutic efficacy of glucose metabolism-modulating strategies. The main approaches include direct targeting and modulation of CAFs or their secreted products. Specifically, selective induction of CAF apoptosis can deplete stromal barriers and facilitate deeper nanomedicine penetration. In addition, CAFs can be reprogrammed into phenotypes that support nanoparticle infiltration and drug diffusion. Strategies targeting CAF-derived products primarily focus on degrading ECM components. For example, by introducing matrix-degrading enzymes or inhibiting ECM crosslinking, both of which enhance nanoparticle penetration and overall treatment efficiency.

Motivated by these insights, a sequential TME and pancreatic ductal adenocarcinoma (PDAC)-targeting nanoplatform (T-AsiG-CPL) was engineered based on cationic liposomes co-encapsulating the nuclear factor kappa-B (NF-κB) inhibitor TPCA-1 and a CD71 ligand-GLUT1 siRNA conjugate (AsiG) via a disulfide linker [180]. TPCA-1 delivery to the TME reprogrammed activated pancreatic stellate cells (PSCs) to a quiescent state, which disrupted their metabolic support of PDAC cells and reduced ECM deposition, thereby enhancing the subsequent uptake of AsiG by tumor cells. AsiG-mediated GLUT1 silencing inhibited aerobic glycolysis in PDAC cells, while TPCA-1-induced NF-κB inhibition attenuated oxidative phosphorylation, synergistically abrogating metabolic crosstalk between PSCs and PDAC cells. In an orthotopic PDAC mouse model, T-AsiG-CPL produced the greatest antitumor effect, reducing tumor weight and volume by 79.13% and 82.79%, respectively, compared with the saline control. In contrast, T-CPL monotherapy achieved only a 66.72% reduction in tumor weight, underscoring the superior efficacy of the combination strategy.

Dense ECM deposition and low intratumoral glucose levels impair immune cell infiltration and effector function, constituting two major barriers to effective antitumor immunotherapy [181, 182]. The chemokine C-X-C motif ligand 1 (CXCL1), frequently overexpressed in various tumors, facilitates tumor progression by activating PSCs and driving TAFs towards a myofibroblastic phenotype. This process results in excessive collagen deposition and formation of a dense, immunosuppressive ECM [183]. To reverse these effects and restore antitumor immunity, mesothelin-targeted nanoparticles (M-s/W-NP) co-loading CXCL1 siRNA and the PI3K inhibitor wortmannin (WT) were engineered [184]. CXCL1 silencing suppressed ECM formation and enhanced immune cell infiltration, while WT downregulated GLUT1 and raised intratumoral glucose levels to support immune function. In an orthotopic pancreatic tumor model, non-targeted nanoparticles carrying both CXCL1 siRNA and WT (I-s/W-NP) showed superior antitumor efficacy compared with WT monotherapy (I-W-NP), indicating a synergistic effect between two agents. Furthermore, the mesothelin-targeted M-s/W-NP exhibited significantly enhanced tumor suppression relative to the non-targeted formulation, underscoring the advantage of active targeting [185]. Moreover, combining M-s/W-NP with anti-PD-L1 antibody produced marked tumor growth inhibition and markedly enhanced responses in immunologically “cold” tumors under checkpoint blockade. However, the complexity of the TME engenders phenotypic diversity and functional heterogeneity among CAF subpopulations, alongside the intricate tumor energy supply, rendering these metabolic intervention strategies challenging [186]. Moreover, the cost-effectiveness and long-term efficacy require further investigation in subsequent studies. 

**Fig. 9:**
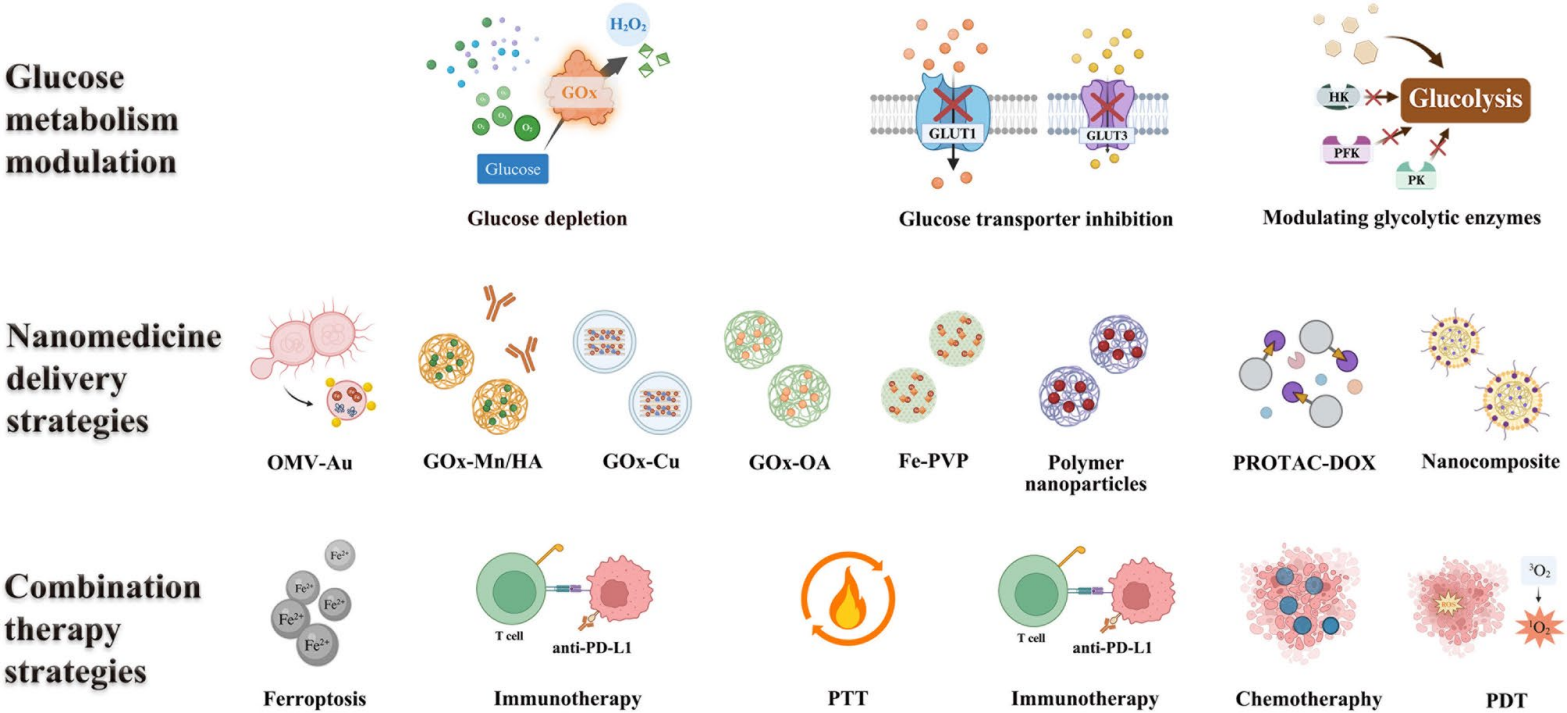
Schematic illustration of how NP-mediated glucose metabolism disruption can be combined with various therapeutic strategies

As illustrated in Fig. 9, leveraging the advantages of co-loading drugs and tumor-targeted distribution, nanomedicines can co-deliver drugs involved in glucose consumption, glycolytic enzyme regulation, and glucose transport inhibition together with chemotherapeutic, radiotherapeutic, or immunotherapeutic drugs, thereby achieving multimodal synergistic antitumor effects. The different therapeutic targets and strategies are summarized in the Table 2.

#### Limitations and challenges

Although matrix modulation can enhance nanoparticle penetration and improve the efficacy of glucose metabolic regulation, excessive disruption of the extracellular matrix may compromise tissue integrity and promote unintended tumor dissemination. Enzymatic degradation of stromal components also carries the risk of off-target damage to normal tissues and blood vessels. Moreover, the dense and heterogeneous architecture of tumor stroma varies significantly among tumor types and disease stages, making it difficult to optimize a uniform matrix-targeting strategy. These challenges highlight the need for spatiotemporally controlled and tumor-selective matrix modulation to balance penetration enhancement with safety

## Conclusion

Targeting glucose metabolism has emerged as a central strategy for disrupting the metabolic foundation that supports tumor growth, treatment resistance, and immune evasion. In this review, we have outlined how nanomedicine enables precise intervention across the glucose metabolic cascade that from glucose uptake and supply, to glycolytic flux, enzymatic regulation, and downstream mitochondrial metabolism. Despite substantial progress, several issues continue to limit translation. For example, tumor metabolic heterogeneity, dynamic metabolic plasticity, and the interplay between cancer cells and stromal components remain major barriers to predictable therapeutic responses. Likewise, the pharmacokinetics, long-term biosafety, and manufacturing consistency of nanomedicines require further validation before clinical deployment. Future work will need to integrate metabolic targeting with immunotherapy, radiotherapy, and regulated cell-death pathways in a more systematic manner, ideally supported by imaging technologies that allow real-time assessment of metabolic states and drug distribution. Overall, glucose-directed nanomedicine is moving toward more rational, mechanism-driven designs that combine metabolic interference with multimodal therapy. Continued progress will depend on deeper mechanistic understanding, improved model systems that better capture human tumor metabolism, and translational strategies that address both biological and regulatory constraints. With these advances, metabolic-targeted nanomedicine holds promise for shaping the next generation of precision cancer therapy.

## Data Availability

No datasets were generated or analysed during the current study.

## Abbreviations

2-DG: 2-deoxyglucose
ABC transporter: TP-binding cassette transporter
ADP: Adenosine diphosphate
AMP: Adenosine monophosphate
ATP: Adenosine triphosphate
BBB: Blood-brain barrier
BSA: Bull serum albumin
CAFs: Cancer-associated fibroblasts
DNA: Deoxyribonucleic acid
DOX: Doxorubicin
ECAR: Extracellular acidification rate
EPR: Enhanced permeability and retention
GCT: γ-glutamyl transpeptidase
GLUT: Glucose transporter
GOx: Glucose oxidase
GSH: Glutathione
H₂O₂: Hydrogen peroxide
HAS: Human bovine serum albumin
HK: Hexokinase
ICIs: Immune checkpoint inhibitors
LDHA: Lactate dehydrogenase A
LM: Liquid metal
O₂: Oxygen
MDSCs: Bone marrow-derived suppressor cells
NADPH: Nicotinamide adenine dinucleotide phosphate
NIR: Near-infrared
OXPHOS: Oxidative phosphorylation
PCD: Programmed cell death
PDT: Photodynamic therapy
PDH: Pyruvate dehydrogenase
PEI: Polyethyleneimine
PFK-1: Phosphofructokinase-1
PKM2: Pyruvate kinase M2
PSCs: Pancreatic stellate cells
PTT: Photothermal therapy
ROS: Reactive oxygen species
TCA: Tricarboxylic acid
TGI: Tumor growth inhibition
TME: Tumor microenvironment
